# Inhibition of macrophage proliferation dominates plaque regression in response to cholesterol lowering

**DOI:** 10.1007/s00395-020-00838-4

**Published:** 2020-12-09

**Authors:** Carmen Härdtner, Jan Kornemann, Katja Krebs, Carolin A. Ehlert, Alina Jander, Jiadai Zou, Christopher Starz, Simon Rauterberg, Diana Sharipova, Bianca Dufner, Natalie Hoppe, Tsai-Sang Dederichs, Florian Willecke, Peter Stachon, Timo Heidt, Dennis Wolf, Constantin von zur Mühlen, Josef Madl, Peter Kohl, Rafael Kaeser, Tobias Boettler, Elsbeth J. Pieterman, Hans M. G. Princen, Benoît Ho-Tin-Noé, Filip K. Swirski, Clinton S. Robbins, Christoph Bode, Andreas Zirlik, Ingo Hilgendorf

**Affiliations:** 1 Department of Cardiology and Angiology I, University Heart Center Freiburg-Bad Krozingen and Faculty of Medicine, University of Freiburg, 55 Hugstetter St, 79106 Freiburg, Germany; 2 Institute for Experimental Cardiovascular Medicine, University Heart Center Freiburg-Bad Krozingen and Faculty of Medicine, University of Freiburg, Freiburg, Germany; 3 Department of Medicine II, Faculty of Medicine, Medical Center–University Freiburg, University of Freiburg, Freiburg, Germany; 4 The Netherlands Organization for Applied Scientific Research (TNO)-Metabolic Health Research, Leiden, Netherlands; 5 INSERM Unit 1148, University Paris Diderot, and Laboratory for Vascular Translational Science, Sorbonne Paris Cité, Paris, France; 6 Center of Systems Biology, Massachusetts General Hospital and Harvard Medical School, Boston, MA USA; 7 Peter Munk Cardiac Centre, University Health Network, Toronto, Canada; 8 Department of Cardiology, University of Graz, Graz, Austria

**Keywords:** Atherosclerosis, Macrophage, Proliferation, Plaque regression

## Abstract

Statins induce plaque regression characterized by reduced macrophage content in humans, but the underlying mechanisms remain speculative. Studying the translational APOE*3-Leiden.CETP mouse model with a humanized lipoprotein metabolism, we find that systemic cholesterol lowering by oral atorvastatin or dietary restriction inhibits monocyte infiltration, and reverses macrophage accumulation in atherosclerotic plaques. Contrary to current believes, none of (1) reduced monocyte influx (studied by cell fate mapping in thorax-shielded irradiation bone marrow chimeras), (2) enhanced macrophage egress (studied by fluorescent bead labeling and transfer), or (3) atorvastatin accumulation in murine or human plaque (assessed by mass spectrometry) could adequately account for the observed loss in macrophage content in plaques that undergo phenotypic regression. Instead, suppression of local proliferation of macrophages dominates phenotypic plaque regression in response to cholesterol lowering: the lower the levels of serum LDL-cholesterol and lipid contents in murine aortic and human carotid artery plaques, the lower the rates of in situ macrophage proliferation. Our study identifies macrophage proliferation as the predominant turnover determinant and an attractive target for inducing plaque regression.

**Supplementary Information:**

The online version contains supplementary material available at 10.1007/s00395-020-00838-4.

## Introduction

International guidelines recommend treating patients with atherosclerotic disease with high-dose statins [20, 41]. For every 40 mg/dL reduction in low-cholesterol diet (LDL) cholesterol by statins the relative risk for major adverse cardiovascular events is curtailed by about 20% [17]. Intravascular imaging studies of atherosclerotic coronaries documented plaque regression with a reduction in plaque volume and macrophage content alongside an increase in fibrous cap thickness following statin treatment [24].

Statins lower LDL-cholesterol levels by inhibiting hydroxy-methylglutaryl-coenzyme A reductase-dependent cholesterol biosynthesis and inducing LDL receptor expression in the liver. LDL-cholesterol lowering also reduces C-reactive protein. These and other findings have fueled the debate as to whether the beneficial effects of statins are primarily attributed to lipid lowering or to pleiotropic, i.e. anti-inflammatory and directly vasoprotective, effects [23, 40]. Multiple in vitro studies documented lipid-independent effects of statins on macrophages and endothelial cells, and statin treatment of Apolipoprotein E (Apoe)-deficient mice limited atherogenesis and monocyte recruitment without affecting cholesterol levels [3, 21, 51, 69]. In contrast, APOE*3Leiden.Cholesteryl ester transfer protein (CETP) mice, a translational mouse model with a humanized lipoprotein metabolism [70], respond to oral statin treatment with (V)LDL-cholesterol lowering, and showed attenuated plaque formation when fed a Western diet [25]. The authors proposed impaired monocyte recruitment into atherosclerotic lesions and suppressed inflammation as underlying mechanisms [26, 64].

While recruited monocytes give rise to plaque macrophages, we showed that local macrophage proliferation dominates cell renewal in established lesions in atherosclerotic mice [49]. Macrophage proliferation in atherosclerotic lesions, particularly in foam cell rich areas, has been described before [19, 45, 50], but its relevance to plaque development was not known. When monocyte production and recruitment are attenuated experimentally, new onset atherogenesis is limited, while interventions in established disease fail to slow plaque progression as lesional macrophages continue to proliferate [29]. Although plaque regression is the ultimate goal in cardiovascular preventive medicine, controversy still surrounds the mechanisms that control the decline in plaque macrophages. Reduction in monocyte influx [13, 42], requirement of differentiation of infiltrating monocytes into reparative macrophages [43], and macrophage emigration [16, 30] were reported to determine plaque regression. In this study, we examined the relative contribution of monocyte infiltration, macrophage proliferation, death and egress in APOE*3Leiden.CETP mice, which model human-like lipid changes in response to oral statin treatment. We show that monocyte-to-macrophage differentiation is a relatively rare event, both in plaque progression and regression, and that the decline in macrophage numbers in regressing plaques mainly results from the suppression of cholesterol-driven local proliferation. Notably, plaque lipid contents and serum cholesterol levels in patients undergoing carotid endarterectomy positively correlated with local macrophage proliferation, supporting the rationale for targeting macrophage proliferation therapeutically.

## Methods

### Animals and diet

Female APOE*3-Leiden.CETP mice were purchased from TNO (Leiden, Netherlands). We used female mice as they are more susceptible to cholesterol-containing diets by having higher plasma cholesterol levels relative to APOE*3-Leiden.CETP males, with established dose-dependent responses to statins [63, 70]. Correspondingly, we purchased female Apoe^*−/−*^ mice (B6.129P2-Apoe^tm1Unc^) from The Jackson Laboratory (Bar Harbor, ME, USA). 8-week-old mice were fed a high-cholesterol diet (HCD, 1.25% w/w cholesterol, D12108 mod., Ssniff GmBH, Soest, Germany) ad libitum for 12 weeks to accelerate atherogenesis, followed by a low-cholesterol diet (LCD, 0.05% w/w cholesterol, semi-synthetic diet T, TNO, Leiden) for 4 weeks to lower proatherogenic plasma cholesterol to levels that allow for normalization through intervention. Next, mice were randomly assigned to up to three groups: 1. continued LCD (control group), 2. LCD supplemented with 0.01% w/w atorvastatin (Pfizer) corresponding to 10 mg/kg body weight per day (statin group), and 3. diet T without cholesterol (free group) for another 4 weeks. To translate the dosing used in our mouse studies (10 mg/kg/d) to human dosing, the following simplified calculation based on body surface area, as accepted by the FDA, can be used as a guide: mouse dose/12.3 × human body weight [36]. Accordingly, 10 mg/kg/day atorvastatin in mice correspond to 65 mg/day for an 80 kg human.

Female Ldlr^–/–^ mice (B6.129S7-Ldlr^tm1Her^/J) were lethally irradiated (10 Gy) and reconstituted with a 1:1 mixture of bone marrow cells from CD45.1 C57Bl/6 (B6.SJL-Ptprc^a^ Pepc^b^/BoyJ) and CD45.2 Msr1^–/–^ (B6.Cg-Msr1^tm1Csk^/J) or CD45.2 CD36^–/–^ (B6.129S1-CD36^tm1Mfe^/J) mice, as previously described [49]. Mice were purchased from The Jackson Laboratory (Bar Harbor, ME, USA). Following 6 weeks of reconstitution consuming chow diet, mice were fed HCD (1.25% w/w cholesterol, D12108 mod., Ssniff GmBH, Soest, Germany) for 12 weeks to induce atherosclerosis.

Mice were housed under specific pathogen-free conditions

### Patients

Twenty-three patients scheduled for elective carotid endarterectomy because of significant carotid artery stenosis gave written informed consent to having their blood and endarterectomy specimens analyzed as approved by the Institutional Review Board of the University Hospital of Freiburg. Blood and tissue samples were collected within 12 h after the last intake of statin drugs if applicable. Detailed patient characteristics are listed in supplemental table 1. Liver tissue biopsies were sampled from three patients during bariatric surgery that had been on oral atorvastatin 40–80 mg/day. Detailed patient characteristics are listed in supplemental table 2.

### In vivo cell labeling

Four weeks into the high-cholesterol diet, 1-μm large yellow-green fluorescent beads (Fluoresbrite YG plain microspheres, Polysciences Inc., Eppelheim, Germany), diluted in 1:4 sterile PBS, were injected intravenously into APOE*3-Leiden.CETP and Apoe^−/−^ mice, respectively, for in vivo cell labeling and tracking of myeloid cells. Fluorescent bead accumulation in the plaque was quantified by immunofluorescence histology. 1 mg bromodeoxyuridine (BrdU) (BD Bioscience, San Jose, CA, USA) was injected intravenously 2 h prior to killing to label proliferating macrophages. BrdU incorporation into CD68^+^ macrophages in the plaque was quantified by immunofluorescence histology.

### Partial body irradiation and bone marrow transfer

Female UBC-GFP mice (C57BL/6-Tg(UBC-GFP)30Scha/J) were purchased from The Jackson Laboratory (Bar Harbor, ME, USA) as donors for bone marrow cell transplantation. APOE*3Leiden.CETP mice with established atherosclerosis following 11 weeks of HCD were lethally irradiated with 10 Gy (RS-2000 Pro Biological System; Rad Source, Buford, GA, USA) while shielding the torso with a lead casing (Belly Shield, Braintree Scientific Inc, Braintree MA, USA, placed on a ¼-inch-thick lead plate) to protect the thoracoabdominal aorta from radiation. 1 × 10^7^ GFP^+^ bone marrow cells were intravenously transplanted into partially irradiated mice.

Modified LDL uptake by bone marrow-derived Mϕ 2 × 10^5^ bone marrow cells were isolated from CD45.1 C57Bl/6 wild-type (WT), CD45.2 Msr1^–/–^ and CD45.2 CD36^–/–^ mice which were used to generate mixed irradiation bone marrow chimeras in Ldlr^–/–^, as described above. Bone marrow cells differentiating into bone marrow-derived Mϕ (BMDM) in the presence of 30 ng/ml colony stimulating factor 1 (Peprotech, Rocky Hill, NJ, USA) over 5 days were stimulated with DiI-medium oxidized LDL (10 μg/ml), DiI-acetylated LDL (1 μg/ml) (Kalen Biomedical, Montgomery Village, MD, USA) or PBS for 4 h before being detached from 24-well plates using trypsin/EDTA. Cells were stained with fixable viability dye (Thermofisher Scientific, Waltham, MA, USA) and anti-F4/80 (Biolegend, San Diego, CA, USA) for flow cytometric quantification of percent viable BMDM and mean fluorescence intensity (MFI) of DiI-labeled modified LDL into the respective cells.

### Histology

Murine aortic roots and arches were embedded in Optimal cutting temperature (OCT) Tissue Tek (Sakura Finetek, Tokyo, Japan) and cut into serial cryostat sections (5 μm) starting at the level of the aortic valve (for root sections). Sections were stained with Oil-red O (Sigma-Aldrich, St. Louis, MO, USA), Masson Trichrome (Sigma-Aldrich, St. Louis, MO, USA), TUNEL (DeadEnd™ Fluorometric TUNEL System, Promega, Mannheim, Germany), anti-CD68 (clone FA-11, BioRad AbD Serotec, Puchheim, Germany), anti-BrdU (GTX128091, GeneTex, Irvine, CA, USA and ab1893, Abcam, Cambridge, UK), anti-GFP (ab290, Abcam, Cambridge, UK). Human carotid endarterectomy specimens were embedded in OCT, frozen, sectioned, permeabilized with 0.1% Triton X-100, and stained with with anti-Ki67 (ab15580, Abcam, Cambridge, UK), anti-CD68 (clone PG-M1, Agilent, Santa Clara, CA, USA), and Hoechst 33342 (Thermofisher Scientific, Waltham, MA, USA). Adjacent slides of the same plaques were stained for Oil-red O (Sigma-Aldrich, St. Louis, MO, USA). Secondary antibodies included rabbit–anti rat biotin conjugated (BA-4001) followed by ImmPACT AMEC Red Substrate (Vector Laboratories, Burlingame, CA, USA), rabbit–anti rat TRITC (PA1-28570, Thermofisher Scientific, Waltham, MA, USA), rabbit–anti rat AF647 (ab169349, Abcam, Cambridge, UK), goat–anti-rabbit (BA-1000) followed by fluorescein avidin DCS (A-2011, Vector Laboratories, Burlingame, CA, USA), donkey-anti-sheep TRITC (ab6897, Abcam, Cambridge, UK), alpaca-anti-rabbit AF488 (ChromoTek, Planegg-Martinsried, Germany), and DAPI Mounting Medium (Carl Roth, Karlsruhe, Germany) according to the manufacturers’ instructions. Images were recorded with the Axioplan 2 and Apotome 2 imaging light-/fluorescence microscope with an AxioCam camera (Carl Zeiss MicroImaging GmbH, Göttingen, Germany) and a confocal Leica TCS SP8 X microscope (Leica Microsystems, Wetzlar, Germany). Images were analyzed with Image Pro Premiere 9.2 (Media Cybernetics, Silver Springs, USA) and Zeiss Zen lite (Carl Zeiss MicroImaging GmbH, Göttingen, Germany).

### Flow cytometry

Murine aortic cells were retrieved through enzymatic digestion with collagenase I, collagenase XI, hyaluronidase, DNAse I and HEPES solution (Sigma-Aldrich, St. Louis, MO, USA) in a thermocycler for 45 min at 750 rpm and 37 °C. Murine blood samples were lysed in RBC lysis buffer (Biolegend, San Diego, CA, USA). Isolated cells from the blood and aorta were counted using a Neubauer chamber (Marienfeld, Lauda-Königshofen, Germany). Cells were stained with specific fluorescent antibodies as indicated (Supplemental Table 3). Ly6C^high^ monocytes were identified as CD45^+^ CD11b^+^, Lin^−^ (Lin = CD3, CD19, NK1.1, Ly6G), Ly6C^high^, CD115^+^, F4/80^low^. Macrophages were identified as CD45^+^ CD11b^+^, Lin^−^, Ly6C^low^, F4/80^high^. Intracellular staining with anti-Ki67 and anti-active Caspase 3, BD Cytoxfix/Cytoperm (#554,722, BD Biosciences, San Diego, CA, USA), BD Perm/Wash (#554,723) and BD Permeabilization Buffer Plus (#561,651) was conducted according to the manufacturer’s instructions. Data were collected on a BD Facs Canto II (BD Bioscience, San Diego, CA, USA) and analyzed with FlowJo (Treestar, Ashland, OR, USA).

### Real-time polymerase chain reaction (PCR)

RNA was extracted from murine aortas using Qiazol and RNeasy Mini Kit (Qiagen, Valencia, CA, USA) according to the manufacturer’s instructions. Quantitative TaqMan-PCR was performed using a Bio-Rad CFX96 Touch Real-Time PCR System and TaqMan probes Mm00443258_m1 (Tnfa), Mm01336189_m1 (IL1ß), Mm00446190_m1 (IL6), Mm00439614_m1 (IL10), Mm01178820_m1 (Tgfb1), Mm01320970_m1 (Vcam1), Mm00441242_m1 (Ccl2). Data were statistically analyzed using the 2^−▵Ct^ method.

### Lipid and enzyme-linked immunosorbent assays

Murine plasma cholesterol and triglyceride levels were measured using cholesterol and triglycerides FS 10′ Multi-purpose kits (DiaSys Diagnostic Systems GmbH, Holzheim, Germany) according to the manufacturer’s instructions. Lipoprotein profiling in murine plasma samples was conducted by LipoSEARCH services (Skylight Biotech Inc., Akita, Japan). Serum Amyloid A levels were measured using a SAA Mouse ELISA Kit (Thermo Fisher Scientific, Waltham, MA, USA), and ApoB levels were measured using the Mouse ApoB ELISA Kit (Abcam, Cambridge, UK) according to the manufacturers’ instructions.

### Mass spectrometry

Plasma and tissue samples were weighed, snap frozen and sent to ImaBiotech SAS (Lille, France) for the quantification of atorvastatin concentration by mass spectrometry. In brief, matrix-assisted laser desorption ionization–Fourier transform ion cyclotron resonance (MALDI–FTICR) was used for imaging mass spectrometry analysis of atorvastatin in murine frozen liver samples sectioned onto indium–tin–oxide glass slides and covered with DAN MALDI matrix (10 mg/ml) in acetonitrile:water 1:1 (v/v). Recordings were made using MALDI–FTICR in negative ionization mode and CASI (continuous accumulation of selected ions) mode centered on *m/z* 564.2917 ± 50 Da mass range with a laser set at 300 shots, 1 kHz to follow atorvastatin in the sections at 200 µm spatial resolution with a SolariX mass spectrometer (Bruker Daltonics, Bremen, Germany). FTMS Control 2.0 and FlexImaging 4.1 software packages (Bruker Daltonics, Bremen, Germany) were used to control the mass spectrometer and set imaging parameters. For liquid tomography–tandem mass spectrometry (LC–MS/MS)-based quantification of atorvastatin, murine tissue samples were extracted with methanol, and murine plasma and human samples were extracted with acetonitrile, and spiked with the internal standard atorvastatin-d5, 1.5 nM. The LC–MS/MS consisted of an UHPLC Ultimate 3000 coupled with TSQ Quantiva (Thermo Scientific, Courtaboeuf, France) equipped with a Cortecs C18 2.7 µm; 2.1 × 30 mm column (Waters, Saint-Quentin-en-Yvelines, France).

### Statistics

Results are presented as mean ± SEM. Differences between two groups were analyzed with unpaired Student’s *T* test or Mann–Whitney test as indicated in the figure legend. To assess differences between more than two groups, one-way ANOVA with Holm–Sidak’s multiple comparisons testing or Kruskal–Wallis with Dunn’s multiple comparisons testing were applied. *p* values ≤ 0.05 denote significant changes. Pearson’s correlation coefficient was used to test for correlation.

## Results

### Oral atorvastatin and diet-induced cholesterol lowering induce phenotypic plaque regression

**Fig. 1:**
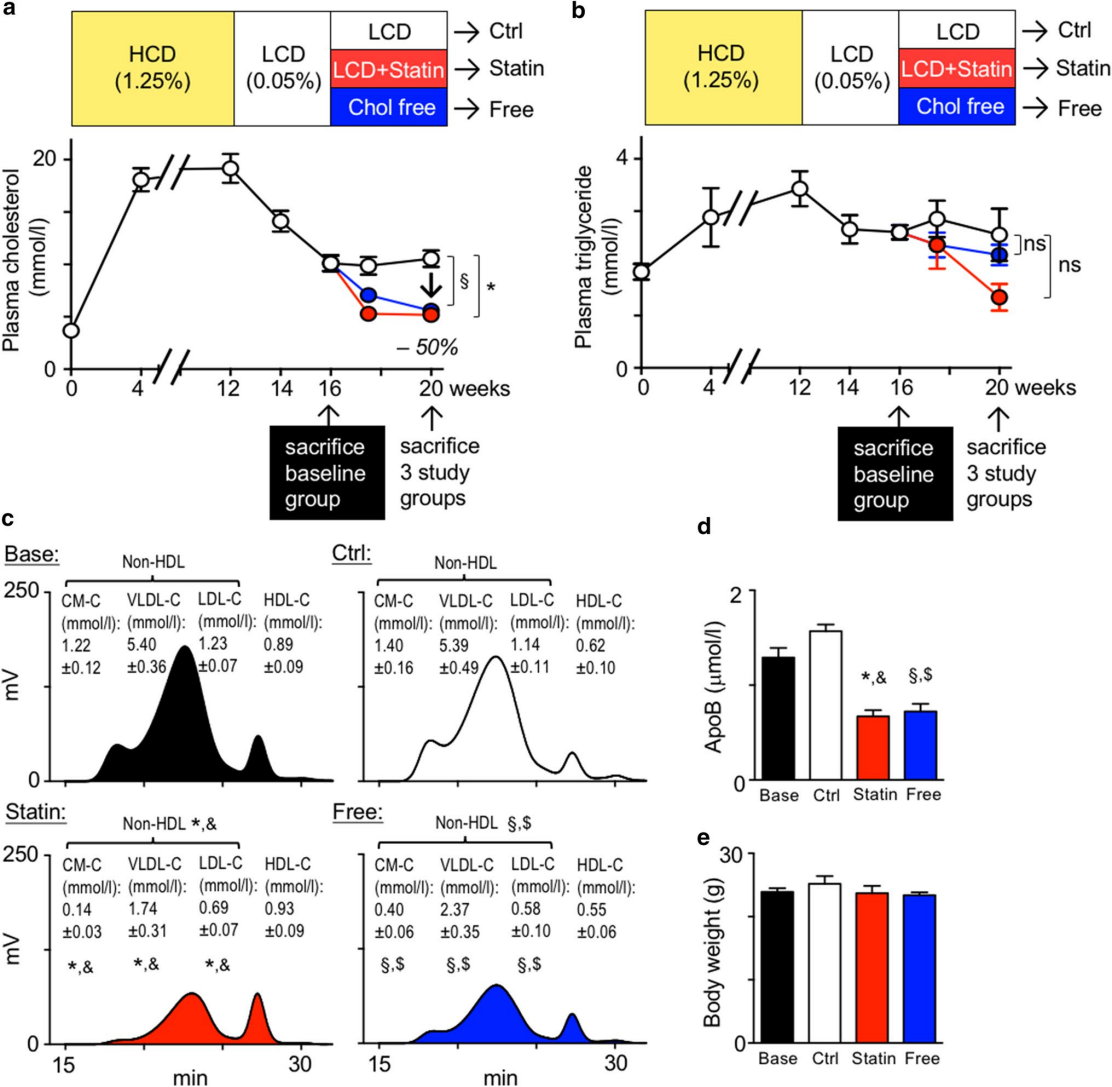
Experimental plaque regression study using atorvastatin and cholesterol-free diet. **a**, **b** Study design and diet scheme depicting plasma total cholesterol (**a**) and triglyceride (**b**) levels measured while feeding APOE*3-Leiden.CETP mice a high-cholesterol diet (HCD, 1.25% w/w cholesterol) for 12 weeks, followed by 4 weeks of low-cholesterol diet (LCD, 0.05% w/w cholesterol). At 16 weeks, the baseline group (Base) was sacrificed and the remaining mice were randomized to three study groups, continued LCD (control, Ctrl), LCD supplemented with 0.01% (w/w) atorvastatin (Statin), and a diet free of cholesterol (Free) for another 4 weeks. Data are presented as mean ± SEM. *,§ *p* < 0.05 denote statistically significant differences between the Control and Statin (*) or Control and Free (§) groups at the time of killing (week 20), *n* = 6 per group, one-way ANOVA. **c** Representative chromatograms and quantification of cholesterol in 4 major lipoprotein classes by gel-filtration HPLC. Data are presented as mean ± SEM. *,&,§,$ *p* < 0.05 denote statistically significant differences between the Base and Statin (&) or Base and Free ($) groups, and between Control and Statin (*) or Control and Free (§) groups at the time of sacrifice, *n* = 6 per group, one-way ANOVA. **d** ApoB plasma levels at the time of sacrifice. Results are presented as mean ± SEM, *,&,§,$ *p* < 0.05 denote statistically significant differences between the Base and Statin (&) or Base and Free ($) groups, and between Control and Statin (*) or Control and Free (§) groups at the time of sacrifice, *n* = 6 per group, one-way ANOVA. **e** Final body weight in all 4 groups at time of killing (Base *n* = 6, *n* = 8 other groups)

To study mechanisms of statin-mediated plaque regression, we induced atherosclerosis in APOE*3-Leiden.CETP mice by feeding a 1.25% cholesterol diet (high-cholesterol diet, HCD) over 12 weeks, accelerating plaque formation, followed by 4 weeks of 0.05% cholesterol diet (low-cholesterol diet, LCD) to lower plasma cholesterol levels to about 10 mmol/L, that would allow for lipid normalization by subsequent therapeutic intervention. At 16 weeks, a baseline group was sacrificed and the remaining mice were randomized to continued LCD that allows for moderate plaque progression, LCD supplemented with 0.01% atorvastatin or a diet free of cholesterol. After 4 weeks, both atorvastatin and the cholesterol-free diet had cut plasma cholesterol levels by half, reaching normolipidemic starting levels. At this point, the three study groups were sacrificed for comparative analysis (Fig. 1a, b). Notably, the 10 mg/kg/d atorvastatin dose in APOE*3-Leiden.CETP mice decreased total and non-HDL cholesterol levels by about 50%, similar to the effects observed in humans taking atorvastatin 40–80 mg per day [53] (Fig. 1a, c). Moreover, atorvastatin or dietary cholesterol restriction significantly reduced circulating ApoB levels (Fig. 1d), a quantitative marker of atherogenic lipoprotein particles, in analogy to changes observed in patients treated with oral atorvastatin [27, 58]. Triglyceride levels fluctuated modestly over the course of diet changes and did not differ between the study groups, unlike cholesterol levels (Fig. 1b). Body weights were similar in all groups (Fig. 1e).

**Fig. 2:**
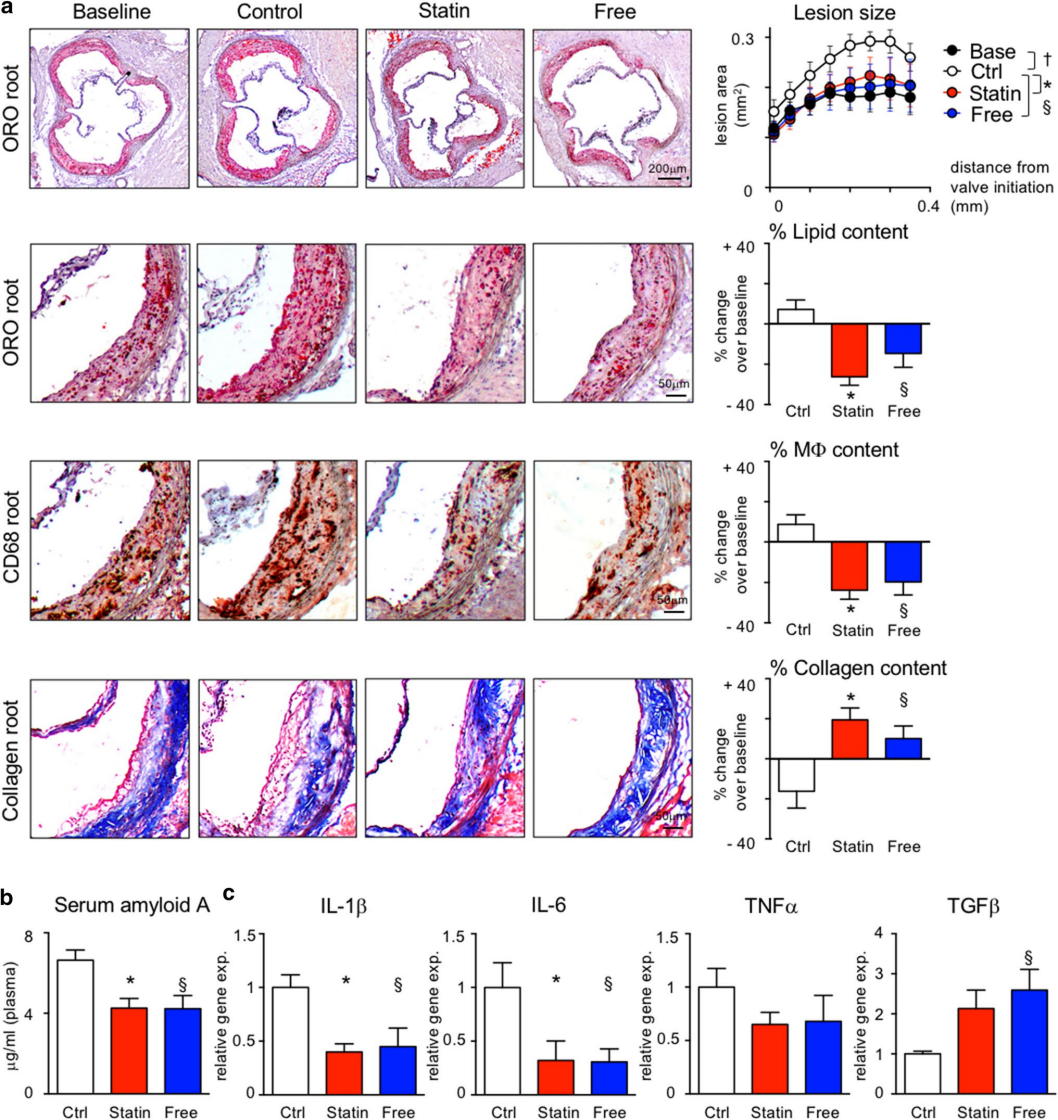
Serum cholesterol lowering leads to phenotypic plaque regression in APOE*3-Leiden.CETP mice. **a** Representative images of aortic root sections stained with Oil-red O (ORO), anti-CD68 (for macrophages, Mϕ) and Masson’s Trichrome (for collagen) on the left. On the right, lesion area was measured on 8 aortic root sections at 50 m intervals starting from valve initiation. Plaque composition was analyzed by quantifying the percent ORO^+^, CD68^+^ and collagen^+^ areas within lesions. Data are presented as mean ± SEM percent change over values in the baseline group to visualize relative changes during plaque progression and regression. *,§,† *p* < 0.05 denote statistically significant differences between the Ctrl and Base (†), Statin (*) and Free (§) groups, respectively, baseline *n* = 6, other 3 groups *n* = 8 per group, one-way ANOVA. *(b)* Serum amyloid A plasma levels at the time of sacrifice. Results are presented as mean ± SEM, *,§*p* < 0.05 denote statistically significant differences between the Ctrl and Statin (*) or Free (§) groups, *n* = 8 per group, one-way ANOVA. **c** Cytokine expression in atherosclerotic aortas. Results are presented as mean ± SEM fold change over control group values, *,§*p* < 0.05 denote statistically significant differences between the Ctrl and Statin (*) or Free (§) groups, *n* = 8 per group, one-way ANOVA

Atherosclerotic lesion size was quantified in the aortic root and arch. Adding atorvastatin to LCD, or feeding a diet free of cholesterol, prevented plaque growth and altered plaque composition. While continued LCD increased lipid and macrophage contents and decreased collagen deposition, atorvastatin and cholesterol-free diets reversed these changes. We refer to these compositional changes as phenotypic plaque regression (Fig. 2a; Supplemental Figs. 1 and 2). As reported previously [64], reducing cholesterol levels by atorvastatin or diet suppresses serum amyloid A levels in plasma, indicative of anti-inflammatory effects (Fig. 2b). Specifically, IL-1β and IL-6 expressions are reduced, and TGFβ is increased in atherosclerotic aortas (Fig. 2c).

### Oral atorvastatin limits monocyte recruitment and macrophage accumulation in atherosclerotic aortas

Quantification of macrophage numbers in enzymatically digested atherosclerotic aortas confirmed a reduction by more than 42% in response to atorvastatin treatment or cholesterol-free diet compared to LCD controls (Supplemental Fig. 3a). Concomitantly, aortic Ly6C^high^ monocyte counts were reduced significantly in atorvastatin-treated mice, and tended to be lower in mice fed a diet free of cholesterol with aortic VCAM-1 and CCL2 expression levels being suppressed (Supplemental Fig. 3a–c). Since the numbers of circulating blood monocytes were similar in all groups we conclude that cholesterol lowering by atorvastatin or dietary restrictions impairs both monocyte infiltration and macrophage accumulation in atherosclerotic lesions (Supplemental Fig. 3a, b and Supplemental Fig. 7a).

**Fig. 3:**
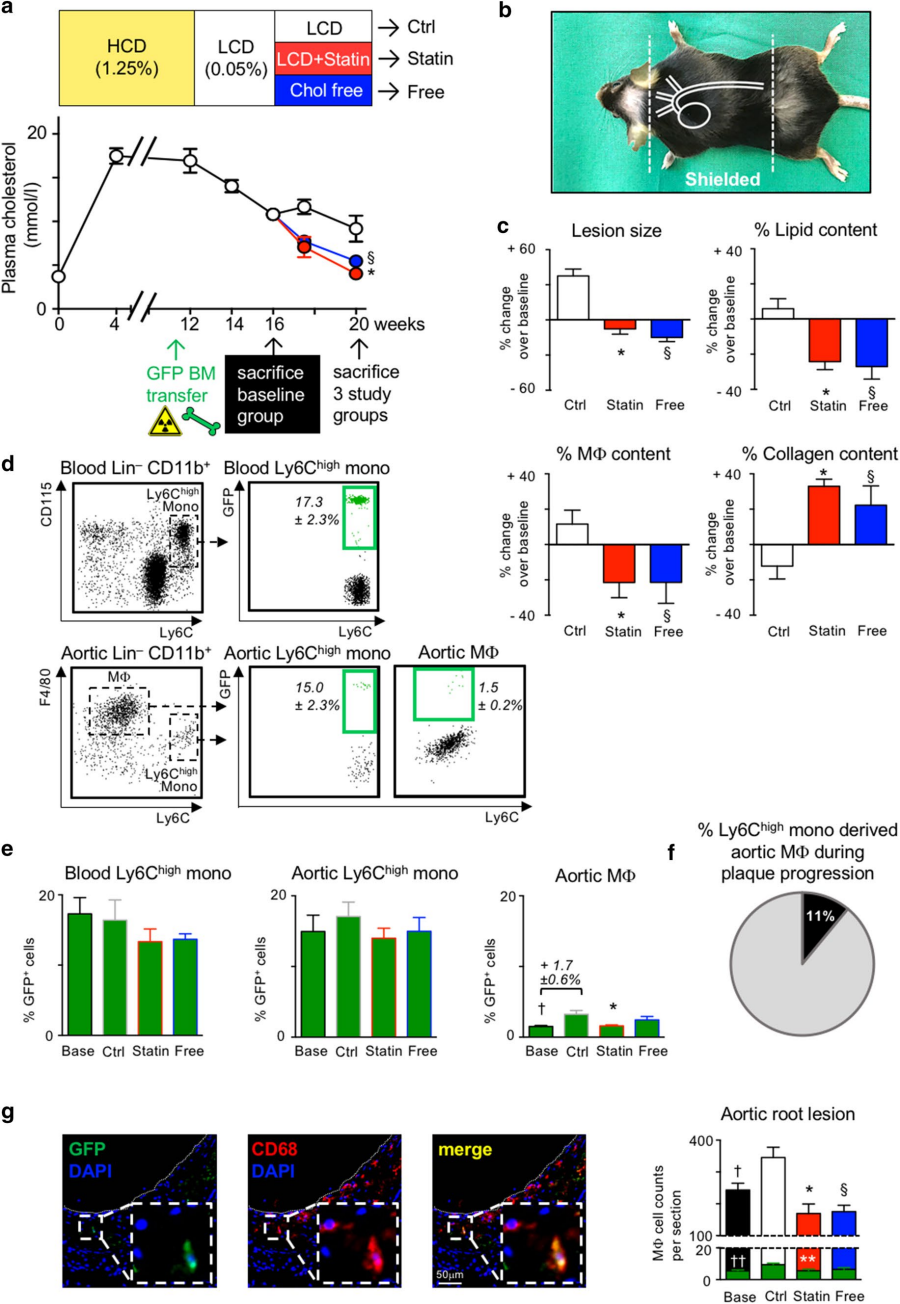
Serum cholesterol lowering reduces lesional macrophage accumulation in established disease, mostly independent of monocyte infiltration in APOE*3-Leiden.CETP mice. **a**, **b** Study design and diet scheme in analogy to Fig. 1a, modified by APOE*3-Leiden.CETP mice undergoing lethal irradiation at week 11 of HCD feeding while shielding the heart and aorta **b** with lead followed by GFP+ bone marrow cell transplantation. Plasma cholesterol levels at indicated time points are presented as mean ± SEM. *,§ *p*  <  0.05 denote statistically significant differences between the Ctrl and Statin (*) or Free (§) groups at the time of sacrifice (week 20), *n*  =  8 per group and time point, one-way ANOVA. **c** Quantification of atherosclerotic lesion size and plaque composition in the aortic root. Data are presented as mean ± SEM percent change over values in the baseline group to visualize relative changes during plaque progression and regression. *,§ *p*  <  0.05 denote statistically significant differences between the Ctrl and Statin (*) or Free (§) groups, baseline *n* =  6, all other 3 groups *n* =  8 per group, one-way ANOVA. Representative images are shown in Supplemental Fig. 4d. **d** Representative images of flow cytometric dot plots showing CD11b+ leukocytes in the aorta and blood at baseline, 5 weeks after GFP+ bone marrow cell transplantation. Data for GFP chimerism among Ly6Chigh monocyte and Mϕ are presented as mean ± SEM. **e** Flow cytometry based quantification of the GFP chimerism among Ly6Chigh monocytes and macrophages (Mϕ) in the blood and aortic cell suspensions, respectively, in all four groups. Results are presented as mean ± SEM, *,† *p*  <  0.05 denote statistically significant differences between the Ctrl and Base (†) or Statin (*) groups, *n*  =  6 (baseline) and n = 8 all other groups, one-way ANOVA. **f** Estimation of the Ly6Chigh monocyte contribution to the pool of aortic Mϕ based on the changes in GFP chimerism during plaque progression (weeks 16–20). **g** Immunofluorescence histology-based quantification of GFP+ (green) cells among all CD68+ Mϕ in aortic root lesions on the right, and representative images of GFP, CD68 and DAPI co-stainings on the left. Results are presented as mean ± SEM, *,§,† *p*  <  0.05 denote statistically significant differences in all CD68+ Mϕ cell numbers between the Ctrl and Statin (*), Free (§) or Base (†) groups. **,††p < 0.05 denote statistically significant differences in GFP+ CD68+ Mϕ cell numbers between the Ctrl and Base (††) or Statin (**)groups, *n*  =  6–8 per group, one-way ANOVA. Representative images for all groups are shown in Supplemental Fig. 4d

Next, we asked whether these two processes were causally linked. To this end, we complemented the feeding and treatment scheme in mice by lethal irradiation before the end of the HCD feeding while shielding the torso to protect the thoracoabdominal aorta from irradiation (Fig. 3a, b). By doing so, Mϕ that had already accumulated in atherosclerotic plaques following 11 weeks of HCD feeding would not be affected by irradiation, preserving natural turnover kinetics. We then transplanted green fluorescent protein (GFP)^+^ bone marrow cells, which seeded in the irradiated, non-protected areas of the body, giving rise to GFP^+^ monocytes and their progeny as a tool to quantify recruitment-dependent and recruitment-independent contributions to Mϕ accumulation in established plaques. One week later, the diet was switched as before to LCD for 4 weeks (Fig. 3a). Meanwhile, a stable GFP cell chimerism of 17.3 ± 2.3% established in circulating Ly6C^high^ monocytes (Fig. 3d,e; Supplemental Fig. 4c). The baseline reference group was killed at week 16 and studied, while the remaining mice were randomized to continued LCD, LCD + 0.01% atorvastatin, or cholesterol-free diet. After 4 weeks of treatment, these three study groups were killed at week 20, and blood and aortas were analyzed with regard to lesion development and GFP cell chimerism. As in the original model (Fig. 1a), both atorvastatin treatment and cholesterol-free diet reduced plasma cholesterol levels by about 50% compared to continued LCD (control) in chimeric mice, while body weights and blood Ly6C^high^ monocyte numbers did not differ between the groups (Fig. 3a; Supplemental Fig. 4a, b). Likewise, histological evaluation of atherosclerotic lesions in the aortic roots and arches replicated the previously observed regression phenotype in irradiated, bone marrow transplanted mice, treated with atorvastatin or fed a diet free of cholesterol (Fig. 3c; Supplemental Figs. 4 and 5). The GFP cell chimerism in Ly6C^high^ monocytes was similar in the blood and aortic tissue of all four groups, indicating that circulating GFP^+^ monocytes infiltrated the atherosclerotic aortas, and that monocyte chimerism remained at equilibrium during the 4-week treatment period (Fig. 3d, e). A GFP chimerism of 15% in aortic Ly6C^high^ monocytes means that for every GFP^+^ monocyte recruited to the aorta, 6 to 7 GFP^–^ endogenous monocytes infiltrated as well (100%/15% = 6.7). If all macrophages in the plaque renewed through replacement by infiltrating monocytes during the 4 weeks of plaque progression (weeks 16 to 20), GFP chimerism among monocytes should translate 1:1 into GFP chimerism among macrophages. However, this was not the case. GFP chimerism in aortic macrophages was only 1/10 of the chimerism in aortic Ly6C^high^ monocytes at baseline (week 16) (Fig. 3d), and although it increased significantly, it did so only by an absolute 1.7 ± 0.6% following 4 weeks of plaque progression with continued LCD (week 20) (Fig. 3e). To estimate the relative contribution of all recruited monocytes to the aortic macrophage pool during plaque progression, accounting for both GFP^+^ and GFP^–^ Ly6C^high^ monocytes that infiltrated, the 1.7% absolute increase in GFP^+^ macrophages over 4 weeks of LCD needs to be multiplied by factor 6.7, resulting in about 11% (Fig. 3f). Although the increase in recruited monocyte-derived macrophages was blunted by atorvastatin treatment in our model of established atherosclerosis, a reduction in macrophage numbers exceeding 11%, such as the > 42% reduction observed with atorvastatin treatment compared to LCD controls (Supplemental Fig. 3a), will mainly depend on local processes of macrophage accumulation that are distinct from and additional to monocyte recruitment and differentiation. Low frequencies of recruited GFP^+^ CD68^+^ macrophages within established atherosclerotic lesions, and the decline in overall macrophage numbers in response to cholesterol lowering, were independently confirmed by histology (Fig. 3g; Supplemental Figs. 4d and 5).

### Oral atorvastatin limits plaque macrophage accumulation predominantly by reducing local macrophage proliferation

**Fig. 4:**
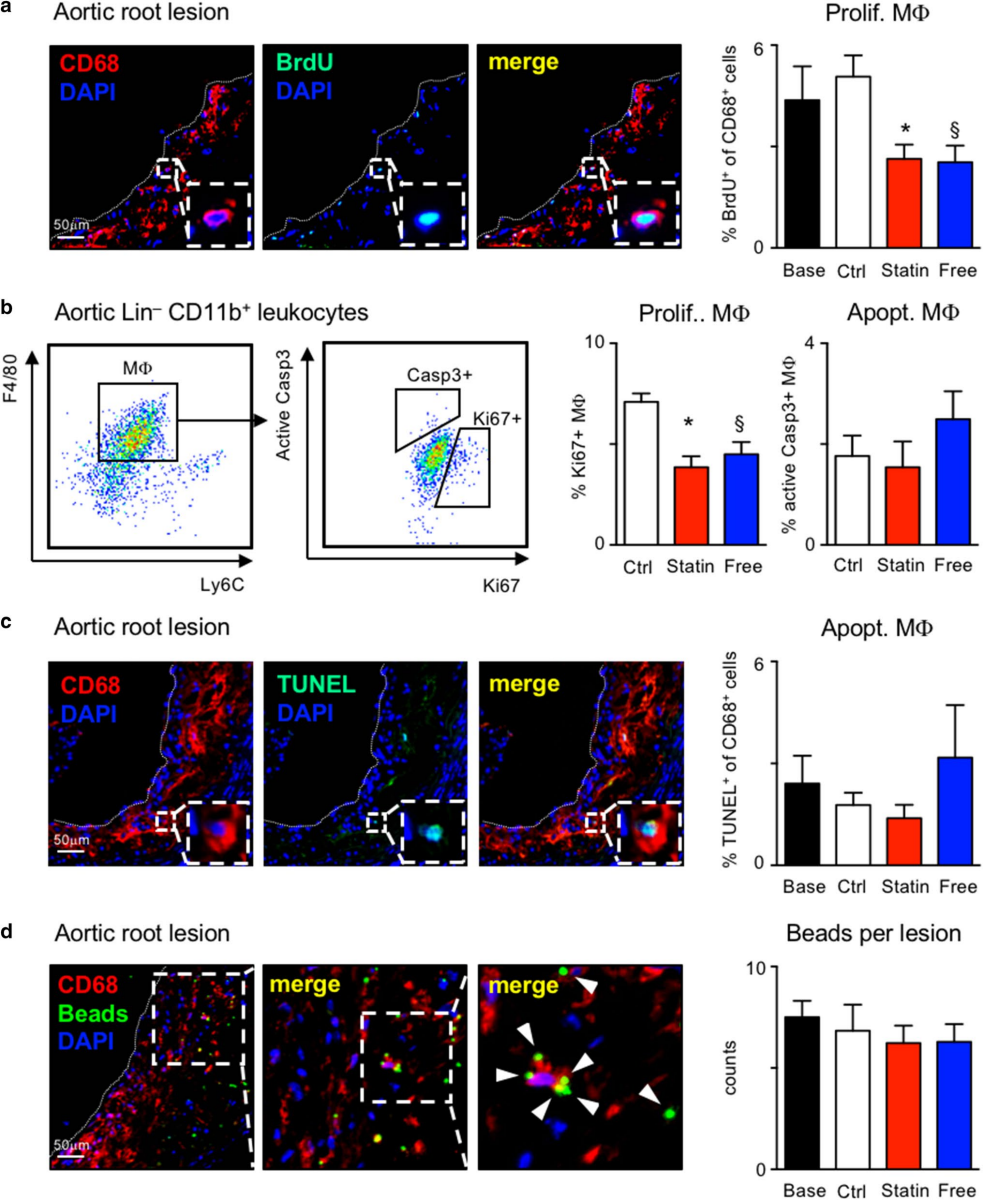
Serum cholesterol lowering inhibits lesional macrophage proliferation in APOE*3-Leiden.CETP mice. **a** Representative images of aortic root lesions stained for CD68, BrdU on the left, and quantification on the right, and **b** representative flow cytometry dot plots (left), and quantification (right) of proliferating aortic Mϕ expressing Ki67^+^ and apoptotic aortic Mϕ expressing active Caspase 3 (Casp3^+^). **c**, **d** Representative images of aortic root lesions stained for CD68, TUNEL **c**, and YG-fluorescent beads **d** on the left, and quantification on the right. Results are presented as mean ± SEM, *,§*p* < 0.05 denote statistically significant difference between the Ctrl and Statin (*) or Free (§) groups, *n* = 8 per group for histology and *n* = 6 per group for flow cytometry readouts, one-way ANOVA

We previously reported that local macrophage proliferation plays a dominant role during plaque progression [49], but its relevance for plaque regression has remained uncertain. Other local processes of cell turnover, such as cell death and egress, were described to influence macrophage content in atherosclerotic lesions [32]. Given that monocyte influx contributes minimally to plaque macrophage kinetics in our model of established atherosclerosis with plaque regression, we decided to compare the frequencies of macrophage proliferation, death and egress in the four study groups. Bromodeoxyuridine (BrdU), a thymidine analog that readily integrates into newly synthesized DNA during the cell cycle, was injected 2 h prior to killing. During this short time period, newly generated monocytes in the bone marrow will not have yet entered blood circulation, and they will, therefore, not contribute to the BrdU^+^ fraction of macrophages in the plaque. Under these conditions, BrdU incorporation into plaque macrophages indicates local proliferation [49]. The proportion of proliferating macrophages was quantified based on immunofluorescent co-staining of anti-BrdU with DAPI and anti-CD68 in aortic root and arch lesions. Macrophage proliferation was reduced to nearly half of time-matched control levels when adding atorvastatin to LCD or switching to cholesterol-free diet (Fig. 4a; Supplemental Figs. 2, 4d, 5, 6a). These data were independently confirmed by flow cytometric intracellular staining for Ki67 in aortic macrophages (Fig. 4b; Supplemental Fig. 7b). Macrophage death was quantified based on TUNEL co-staining with DAPI and anti-CD68 in aortic root and arch lesions, and flow cytometric intracellular staining for active caspase 3 (Fig. 4b, c; Supplemental Figs. 2 and 7b). We observed no difference between the groups in either test. Potential macrophage egress was determined using fluorescent bead labeling, as previously described [16, 29, 55, 67]. Fluorescent beads were injected early during atherogenesis after 4 weeks of HCD feeding (Supplemental Fig.6d). Within 7 days, these beads were cleared from the blood stream by circulating monocytes and neutrophils (Supplemental Fig. 6e) that infiltrate the nascent plaques. Since monocytes and neutrophils are short lived, beads that do not immediately leave the plaque within the cells that brought them in remain inside monocyte-derived macrophages. Alternatively, beads are disposed and trapped in the plaque when bead-laden cells die, until being taken up by other phagocytes. Only as bead-laden phagocytes emigrate from the plaque, the number of beads inside the plaque can decrease over time. Therefore, the total number of beads that are retained in the plaques is inversely proportional to the number of macrophages that might exit the plaque. We did not observe a significant decline in plaque bead content over the 4-week time period of plaque regression (Fig. 4d; Supplemental Figs. 2 and 6c), indicating that macrophage egress is unlikely to be a major contributor to the loss of macrophages with oral atorvastatin treatment. Taken together, our data show that suppression of local macrophage proliferation in the presence of unchanged cell death mediates atorvastatin induced plaque regression.

### Reduced macrophage proliferation depends on cholesterol lowering in mice

We wondered whether the observed effects of oral atorvastatin treatment were primarily mediated by cholesterol lowering or by so-called pleiotropic effects. Phenotypically, in the two APOE*3-Leiden.CETP mouse models, with and without partial body irradiation, both drug-induced and diet-mediated normalization of cholesterol levels resulted in similar plaque regression (Figs. 1a, 2a 3a–c Supplemental Figs. 2, 4d, 5). Mechanistically, both interventions suppressed local macrophage proliferation (Fig. 4a and Supplemental Figs. 2, 4d, 5 and 6a), suggesting that the cholesterol lowering effects matter. In addition, we quantified atorvastatin drug levels by mass spectrometry in livers, plasma and atherosclerotic aortas of APOE*3Leiden.CETP mice fed LCD + 0.01% atorvastatin. We detected high levels of atorvastatin in livers (59 ng/g) and lower levels in plasma (1.9 ng/ml), but virtually none in aortic tissues, arguing against atorvastatin affecting plaque macrophages directly when administered orally (Supplemental Fig. 8a, b).

**Fig. 5:**
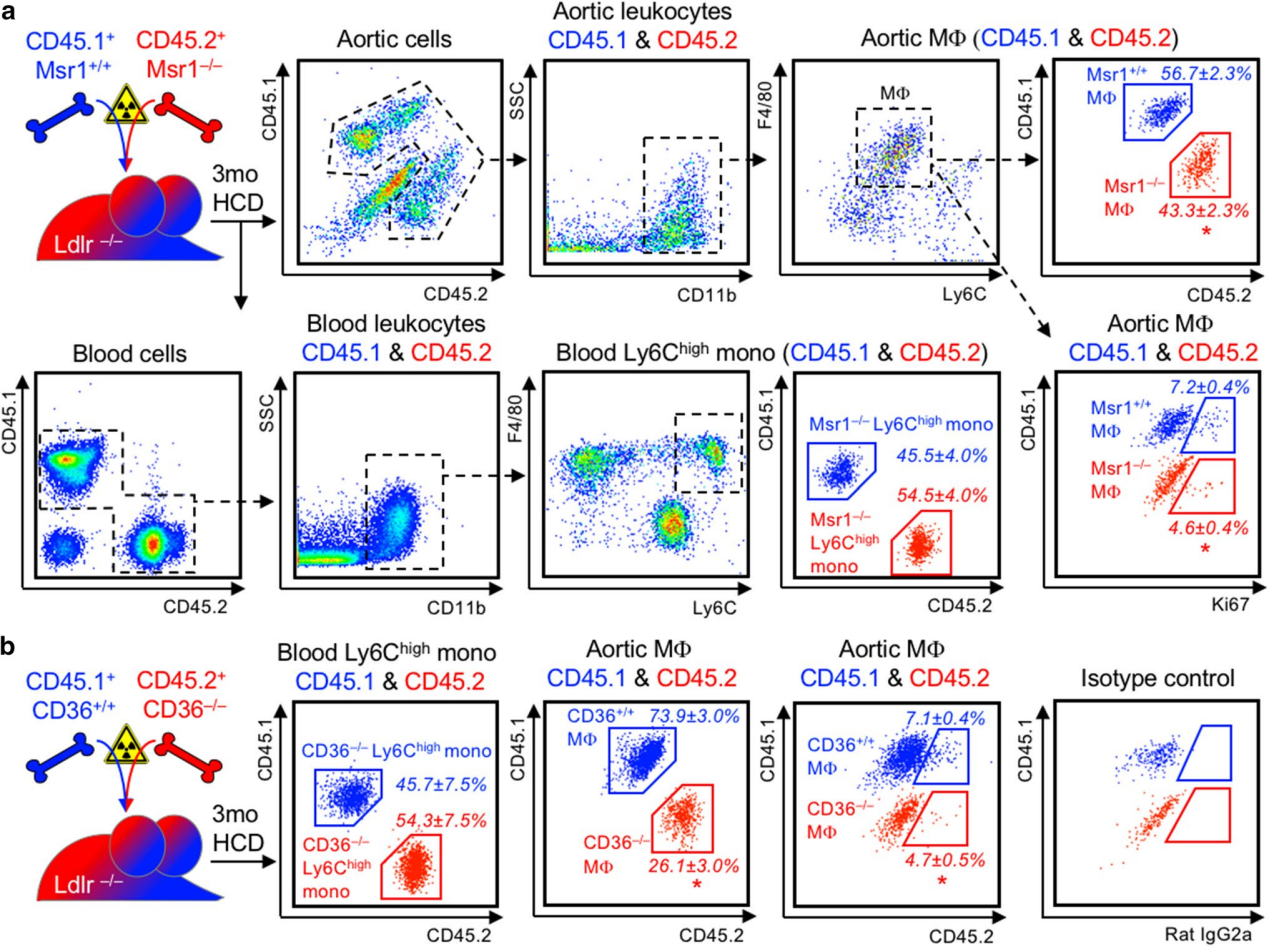
Cholesterol-rich modified LDL-uptake-mediating scavenger receptors Msr1 and CD36 directly propagate MF proliferation in plaques. **a** Ldlr^–/–^ mice were irradiated and reconstituted with a 1:1 mixture of CD45.1 Msr1^+/+^ and CD45.2 Msr1^–/–^ bone marrow cells for 6 weeks before starting a high-cholesterol diet (HCD) for 3 months. Msr1^+/+^ and Msr1^–/–^ Ly6C^high^ monocytes (mono) in blood and macrophages (Mϕ) in enzymatically digested atherosclerotic aortas were distinguished based on exclusive CD45.1 and CD45.2 expression, as depicted in the representative dot plots and gating strategies. The chimerism of Msr1^+/+^ and Msr1^–/–^ within blood Ly6C^high^ monocytes and aortic Mϕ was quantified based on CD45.1 and CD45.2 staining, and the fraction of proliferating cells was assessed based on intracellular Ki67 staining in Msr1^+/+^ and Msr1^–/–^ MF. Results are presented as mean percent ± SEM cell chimerism and Ki67^+^ fraction of the respective population, *n* = 4 per group, *p < 0.05 denotes statistically significant differences between Msr1^+/+^ and Msr1^–/–^ Ly6C^high^ monocyte or Mϕ population, Mann–Whitney test. **b** Mixed CD45.1 CD36^+/+^ and CD45.2 CD36^–/–^irradiation bone marrow chimeras were generated in Ldlr^–/–^ mice and analyzed in analogy to Msr1^+/+^/Msr1^–/–^ chimeras, as described above. Results are presented as mean percent ± SEM cell chimerism and Ki67^+^ fraction of the respective population, *n* = 4 per group, *p < 0.05 denotes statistically significant differences between CD36^+/+^ and CD36^–/–^ Ly6C^high^ monocyte or Mϕ population, Mann–Whitney test

Given that oral atorvastatin reduces the number of circulating ApoB-lipoprotein particles and their cholesterol contents (Fig. 1a, c, d), we asked instead, whether the uptake of cholesterol-rich modified LDL into plaque macrophages will influence proliferation, directly. To this end, we generated mixed-bone marrow chimeras in irradiated Ldlr^–/–^ mice reconstituted with a mixture of macrophage scavenger receptor 1 (Msr1) deficient and competent bone marrow (Fig. 5a). Msr1^+/+^ bone marrow cells were isolated from CD45.1 C57Bl/6 mice to distinguish wild-type from knockout cells in the reconstituted recipients according to exclusive CD45.1 or CD45.2 expression. In analogy, we generated mixed Ldlr–/– bone marrow chimeras with CD45.1 CD36^+/+^ and CD45.2 CD36^–/–^ cells (Fig. 5b). Msr1 preferentially mediates the uptake of LDL modified by acetylation into macrophages, whereas oxidized LDL is preferentially engulfed via CD36 (Supplemental Fig. 9a). Following 3 months of HCD feeding, atherosclerosis developed (Supplemental Fig. 9b, c), and blood and aortas were analyzed by flow cytometry for CD45.1/CD45.2 chimerism and differences in the expression of proliferation marker Ki67 in CD45.1 Msr1^+/+^ versus CD45.2 Msr1^–/–^ macrophages, and in CD45.1 CD36^+/+^ versus CD45.2 CD36^–/–^ macrophages, respectively. Proliferation of Msr1- or CD36-deficient macrophages was reduced by about 1/3 compared to wild-type (WT) macrophages in the same plaque exposed to the same lipids and external stimuli (Fig. 5a, b). The proliferative advantage of WT macrophages over knockout macrophages may have contributed to the shift in chimerism towards CD45.1 WT macrophages observed in atherosclerotic aortas.

**Fig. 6:**
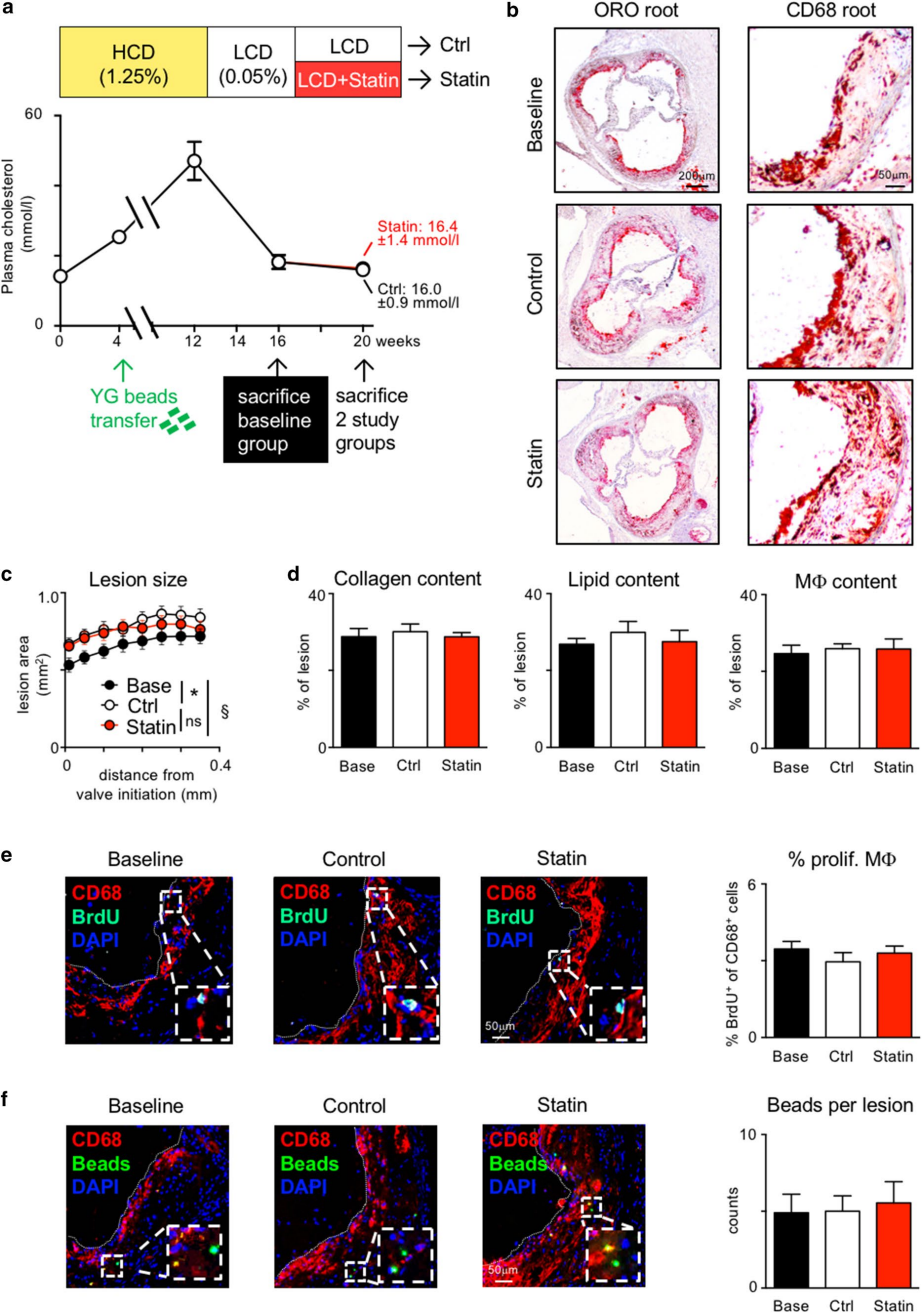
Atorvastatin fails to induce plaque regression without cholesterol lowering in Apoe^–/–^ mice. **a** Study design and diet scheme similar to Fig. 1a with Apoe^–/–^ mice fed a high-cholesterol diet (1.25% cholesterol) for 12 weeks, followed by 4 weeks of low-cholesterol diet (LCD, 0.05% cholesterol) (Baseline, Base), and 4 weeks of continued LCD (Control, Ctrl) versus LCD supplemented with 0.01% (w/w) atorvastatin (Statin). **b**–**d** Lesion size was measured on 8 aortic root sections at 50-µm intervals starting from valve initiation **c**, and plaque composition was analyzed by quantifying the percent ORO^+^, CD68^+^ and collagen^+^ areas within lesions (d). Representative images of aortic root sections are show in (b) and Supplemental Fig. 8. Data are presented as mean ± SEM. *,§*p* < 0.05 denote statistically significant differences between the Ctrl and Base (*) or Statin (§) groups, Base group *n* = 7, Ctrl and Statin groups *n* = 8 each, one-way ANOVA. **e**, **f** Quantification of Mϕ proliferation based on BrdU incorporation **e** and of Mϕ egress based on bead retention **f** in aortic root lesions. Results are presented as mean ± SEM on the right, Base group *n* = 7, Ctrl and Statin groups *n* = 8 each, one-way ANOVA. Representative images are shown on the left

Finally, we treated Apoe^–/–^ mice with established atherosclerosis in analogy to the feeding study in APOE*3-Leiden.CETP mice comparing LCD with and without supplementation of 0.01% atorvastatin (Fig. 6a). As previously reported [6, 34, 35, 39], oral atorvastatin at this dose range did not reduce systemic cholesterol levels, weight gain or monocytosis in Apoe^–/–^ mice (Fig. 6a; Supplemental Fig. 8c, d). Consequently, plaques continued to grow instead of regressing under atorvastatin treatment (Fig. 6b–d; Supplemental Fig. 8e). In the absence of a cholesterol lowering effect in Apoe^–/–^ mice, atorvastatin did not alter macrophage accumulation, proliferation, death and retention in atherosclerotic lesions (Fig. 6e, f; Supplemental Fig. 8f, g). Taken together, these data suggest that atorvastatin inhibits plaque macrophage proliferation indirectly via lowering of cholesterol and ApoB-lipoprotein levels.

### Plaque macrophage proliferation in humans correlates with serum LDL-cholesterol and plaque lipid content

**Fig. 7:**
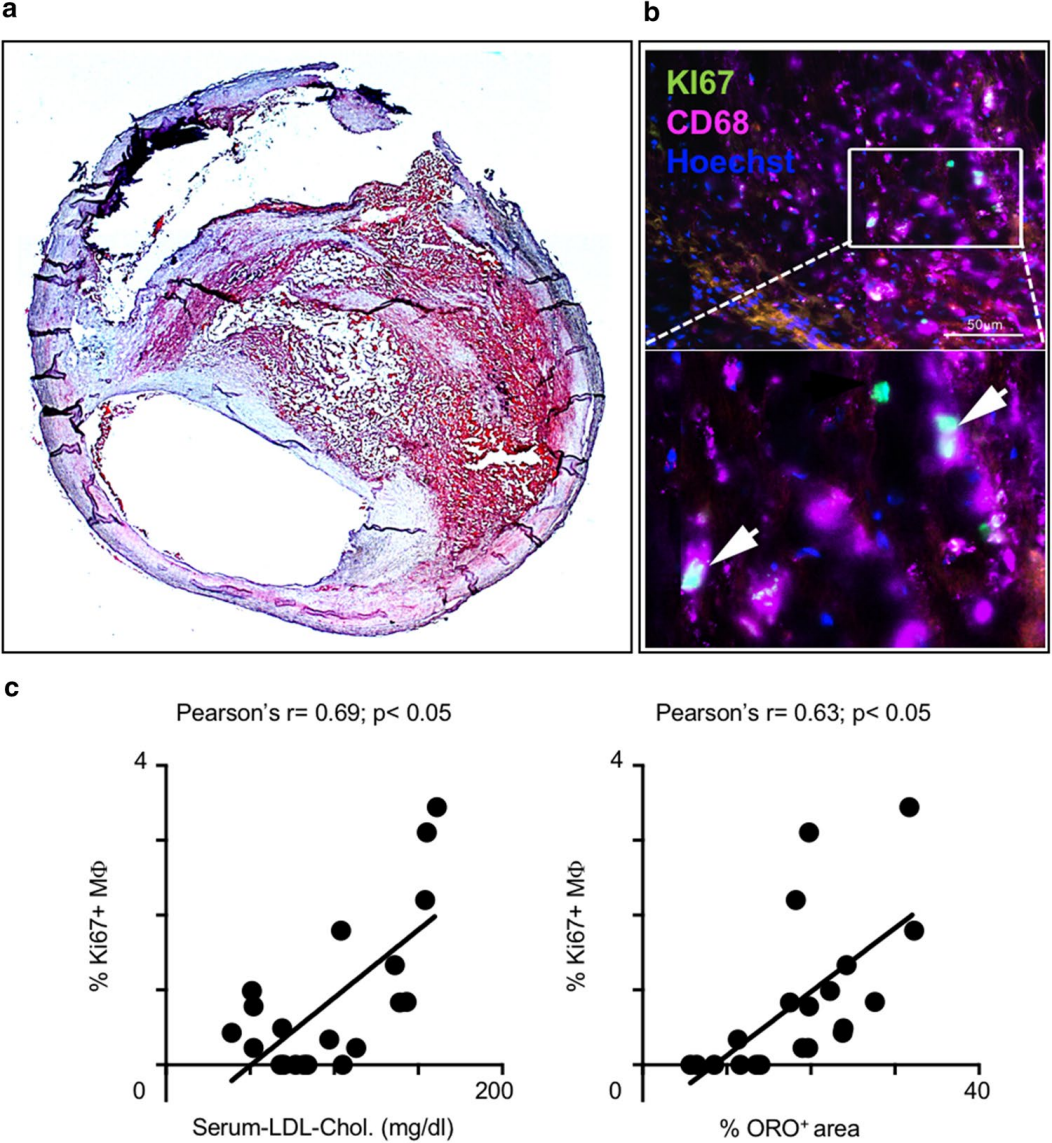
LDL-cholesterol correlates with local macrophage proliferation in human atherosclerotic plaques. **a** Representative image of a carotid artery section with plaque, where lipids are stained with Oil-red O. **b** Representative images of CD68 (Mϕ, KI67 (proliferation), Hoechst (nuclei) co-staining in the plaque. Arrows mark triple-positive, proliferating Mϕ** c** Correlation of plaque Mϕ proliferation rate with serum LDL-cholesterol levels and plaque lipid content (ORO^+^ area), respectively. Each point represents an individual patient (*n* = 20), *r*: Pearson’s correlation coefficient, *p* < 0.05 denotes significant correlation

Next, we asked whether the link between cholesterol levels and macrophage proliferation in atherosclerotic lesions also applied to human plaques. To this end, we collected blood and plaque tissue samples from 23 patients undergoing carotid endarterectomy (Supplemental Table 1). All but three patients were taking statins in line with guideline recommendations. Still, serum LDL-cholesterol levels ranged from 32 mg/dl to 161 mg/dl. Of note, atorvastatin plasma levels in patients corresponded to those measured in our APOE*3-Leiden.CETP mouse model, and, unlike the liver, atorvastatin was hardly detectable in human plaque tissues (Supplemental Fig. 8b, Supplemental Table 2). Macrophage proliferation was assessed by co-staining DAPI, anti-CD68 and anti-Ki67 (Fig. 7b). In adjacent slides, plaque lipid content was determined based on Oil-red O staining (Fig. 7a). Macrophage proliferation correlated positively with both serum LDL-cholesterol levels (*r* = 0.69) and plaque lipid contents (*r* = 0.63) (Fig. 7c).

## Discussion

We set out to investigate the cellular processes that determine plaque regression during statin treatment. Plaque progression depends on macrophage accumulation, and, conversely, macrophages vanish during plaque regression [16, 60]. Multiple processes determine macrophage accumulation in the plaque, including monocyte recruitment and differentiation, macrophage proliferation, death and egress [32]. Macrophages of prenatal origin reside in the adventitia and self-sustain through proliferation into adulthood largely independent of monocyte recruitment [15, 65]. Whether these embryonically derived macrophages in the adventitia directly contribute to macrophages accumulating in intimal lesions is unknown. A small population of intima resident macrophages shows limited proliferation capacity and is marginalized during plaque progression [68] as monocytes infiltrate giving rise to proliferating macrophages [49].

Previous experimental studies in atherosclerotic mice showed that both macrophage accumulation and plaque growth are limited by statin treatment. Most of these studies were conducted in Apoe^–/–^ and Ldlr^–/–^ mice, which do not respond to statins with a significant reduction in serum LDL-cholesterol at clinically relevant doses [6, 10, 34, 35]. This was interpreted as a proof of pleiotropic drug effects. Protective mechanisms proposed included suppression of endothelial cell adhesion molecule expression, stimulation of endothelial nitric oxide synthase and inhibition of leukocyte integrin LFA1 [2, 40, 66]. As a result of these molecular effects, monocyte recruitment to atherosclerotic lesions would be reduced, a finding we observed in our study as well. It was thought that impaired monocyte infiltration limited macrophage accumulation in plaques following statin treatment, but this has not been tested. Our model of irradiating mice with established atherosclerosis and aortic shielding followed by GFP^+^ bone marrow transplantation, allowed us to quantify the contribution of blood monocytes to the plaque macrophage pool with and without cholesterol lowering. We calculated that during 4 weeks of plaque progression, only 11% of macrophages derived from newly recruited monocytes. These numbers matched our previous estimate of 13% monocyte contribution to the macrophage pool, based on parabiosis and BrdU incorporation in Apoe^–/–^ mice [49]. Thus, inhibition of monocyte recruitment, as observed with atorvastatin, could not fully explain the large reduction in macrophage numbers by 40–50% in atherosclerotic lesions following atorvastatin treatment.

We, therefore, investigated cellular processes localized within the plaque, i.e. macrophage proliferation, death or egress. Indeed, macrophage proliferation, as determined by intracellular Ki67 staining and BrdU incorporation, was reduced by almost 50% by atorvastatin treatment. A previous study reported that lipophilic simvastatin, incorporated into a synthetic HDL particle, invaded the plaque, locally inhibiting macrophage proliferation and decreasing macrophage numbers in Apoe^–/–^ mice, without affecting serum cholesterol levels [14, 59]. Multiple in vitro studies also documented direct anti-inflammatory and anti-proliferative effects of statins on macrophages in culture [5, 52, 62]. When we treated APOE*3-Leiden.CETP mice with oral atorvastatin, achieving plasma drug levels comparable to those in patients treated with 40–80 mg atorvastatin per day, the drug was not detected in atherosclerotic arteries, unlike the nanoparticle approach. In line, atorvastatin was also hardly detectable in human carotid artery plaques. The absence of drug accumulation in plaques does not formally exclude the possibility of local pharmacological effects. However, two additional findings in our study argue against relevant direct pleiotropic statin effects on macrophage proliferation within the plaque. First, diet induced plasma cholesterol lowering, alone, to levels achieved with oral atorvastatin treatment in APOE*3-Leiden.CETP mice yielded similar results with regard to plaque regression, dampening of systemic and local inflammation, and inhibition of macrophage proliferation in the plaque. Second, when we treated atherosclerotic Apoe^–/–^ mice with oral atorvastatin, plasma cholesterol levels remained elevated, and macrophage counts and proliferation rates were unaffected. This finding is in accordance with a previous observation we made when inhibition of monocyte production, lesion infiltration and differentiation in Apoe^–/–^ mice with established atherosclerosis failed to slow plaque progression and accumulation of macrophages, which continued to proliferate in situ [29]. When we deleted modified lipoprotein uptake-mediating scavenger receptors (Msr1 or CD36) in macrophages accumulating in murine atherosclerotic aortas next to scavenger receptor expressing macrophages, their proliferation was relatively suppressed. These data support our hypothesis that the uptake of cholesterol-rich modified lipoproteins stimulates macrophage proliferation in atherosclerotic lesions, directly, but the intracellular signaling pathways remain to be determined. Notably, intracellular cholesterol was also found to stimulate hematopoietic stem and progenitor cell proliferation in atherosclerotic mice [33]. Oxidized LDL provokes colony-stimulating factor 1 (Csf1) secretion by endothelial cells, for example [44]. A recent study described increased macrophage survival and proliferation in the plaque in response to Csf1 production by endothelial cells and vascular smooth muscle cells, in particular [57]. This may represent an additional, indirect mechanism by which statins, via systemic reduction in cholesterol-rich LDL particle numbers, inhibit lesional macrophage proliferation.

In support of our lipid uptake hypothesis, a recent experimental study comparing gene expression profiles of lipid-rich and lipid-poor macrophages from atherosclerotic murine aortas reported (in the supplement) that proliferation marker Ki67 expression was almost doubled in lipid-rich cells [22]. According to a recent meta-analysis of leukocyte diversity in atherosclerotic mouse aortas, based on single-cell RNA sequencing, these lipid-rich foamy macrophages correspond to the Trem2 (triggering receptor expressed on myeloid cells-2) macrophage subset specialized in lipid metabolism, distinct from inflammatory, resident-like and interferon-inducible macrophage subsets [71]. These macrophage populations partially overlap with subsets identified in human plaques [12, 18]. Ingenuity pathway analysis of a single cell dataset of aortic macrophages isolated from Apoe^–/–^ mice described enrichment of proliferative, survival and motility genes in one of four macrophage clusters, as opposed to inflammation, apoptosis and phagocytosis related genes in the other clusters [31]. While these transcriptional data substantiate the presence of proliferating macrophages in atherosclerotic aortas, they do not inform on whether all subsets of lesional macrophages are equally prone to undergo cell cycling in situ. The plasticity of lesional macrophages is the focus of ongoing research. Macrophage-like cells that express macrophage markers such as CD68 and Lgals3, but not leukocyte marker CD45, may arise from vascular smooth muscle cells [54], which clonally expand in the plaque [9]. They are reported to account for 16–30% of macrophage marker-positive cells in the plaque [1, 54]. In our study in APOE*3-Leiden.CETP mice, lowering of cholesterol and atherogenic ApoB-lipoprotein levels reduced proliferation of CD68 expressing cells in general in the plaque, and this may affect macrophages of both leukocyte and vascular smooth muscle cell origin.

The relevance of macrophage egress for plaque regression is controversially debated with several papers arguing against egress [28, 29, 42, 67] and a number of papers that argue for egress [16, 30, 55]. The discrepancies may arise from differences in the models used to induce normolipidemia and timing related to bead transfer. In our models of drug- and diet-induced cholesterol lowering in APOE*3-Leiden.CETP mice, and of statin treatment of Apoe^–/–^ mice over 4 weeks, we found no indication for significant macrophage egress from established plaques, in line with the aforementioned papers.

Translating our experimental findings to humans, we showed that the levels of serum cholesterol lowering achieved in patients undergoing carotid endarterectomy is inversely correlated with the frequency of plaque macrophage proliferation. The more lipids one detects in the plaque, the higher the proportion of proliferating macrophages in situ. Studying the effects of statins, the most widely used and potent drugs in cardiovascular secondary prevention, our work documents the importance of local macrophage proliferation for plaque progression and lipid therapy-based regression. While oral atorvastatin inhibits macrophage proliferation indirectly via systemic cholesterol and ApoB-lipoprotein reductions, direct targeting of macrophage proliferation may emerge as a potent add-on therapy to support plaque regression. Anti-proliferative drugs such as paclitaxel and methotrexate or mTOR-inhibitor rapamycin, incorporated into a lipid nanoparticle LDE resembling low-density lipoproteins or biomimetic nanoparticles, induced plaque regression and loss of plaque macrophages in murine and rabbit models of atherosclerosis with limited systemic toxicity [4, 7, 11]. More recently, LDE-paclitaxel was injected into eight patients with aortic atherosclerosis six times every 3 weeks, and computer tomography images of the atherosclerotic aortas were compared before and after 1–2 months of treatment with those obtained in untreated patients. Remarkably, half of the treated patients showed a reduction in plaque size without significant changes to their blood cell counts, while at the same time all the nine untreated patients showed mild disease progression [56]. A number of clinical trials on treating inflammation in atherosclerosis have recently been published or are ongoing. While colchicine and canakinumab appear to protect from atherosclerotic complications post myocardial infarction [37, 38, 47, 48, 61], low-dose methotrexate failed to do so in the latest CIRT trial [46]. Of note, at low doses and with folic acid supplementation, methotrexate does not interfere with DNA synthesis, but it may suppress inflammation via the release of adenosine [8], although no signs of modulating inflammation were seen in the CIRT trial. Whether interleukin-1β blockade and colchicine treatment influence plaque macrophage proliferation remains to be determined. We are finally entering an era where our conceptual understanding of the role of inflammation in atherosclerosis derived from numerous preclinical studies translates into clinics. Our study fits into this picture by identifying macrophage proliferation as a relevant and modifiable determinant of inflammatory cell accumulation in atherosclerotic lesions to be targeted therapeutically in support of plaque regression.

## Supplementary Information

Below is the link to the electronic supplementary material.

Supplementary file1 (PDF 5119 KB)

## Data Availability

Data are available from the corresponding author upon reasonable request.
