# Supplementary material for: Inhibition of macrophage proliferation dominates plaque regression in response to cholesterol lowering
Source: Basic Res Cardiol. 2020 Dec 9;115(6):78. doi: 10.1007/s00395-020-00838-4 (PMC7725697; doi:10.1007/s00395-020-00838-4)
Supplement: Supplementary file 1 — Supplementary file1 (PDF 5119 KB) [file 395_2020_838_MOESM1_ESM.pdf]

Supplemental Material

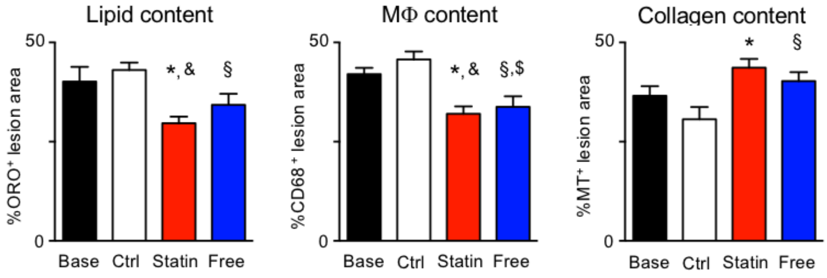

Supplemental Fig. 1. Plaque composition changes in aortic roots of APOE*3-Leiden.CETP mice. Quantification of plaque lipid (ORO), MF (CD68) and collagen (Masson trichrome) content as percentage of lesion area. Data are presented as mean ± SEM. *,§,&,$ $p<0.05$ denote statistically significant differences between the Ctrl and Statin (*) or Free (§) groups, and between Base and Statin (&) or Free ($) group, respectively, baseline n=6, all other 3 groups n=8 per group, one-way ANOVA.

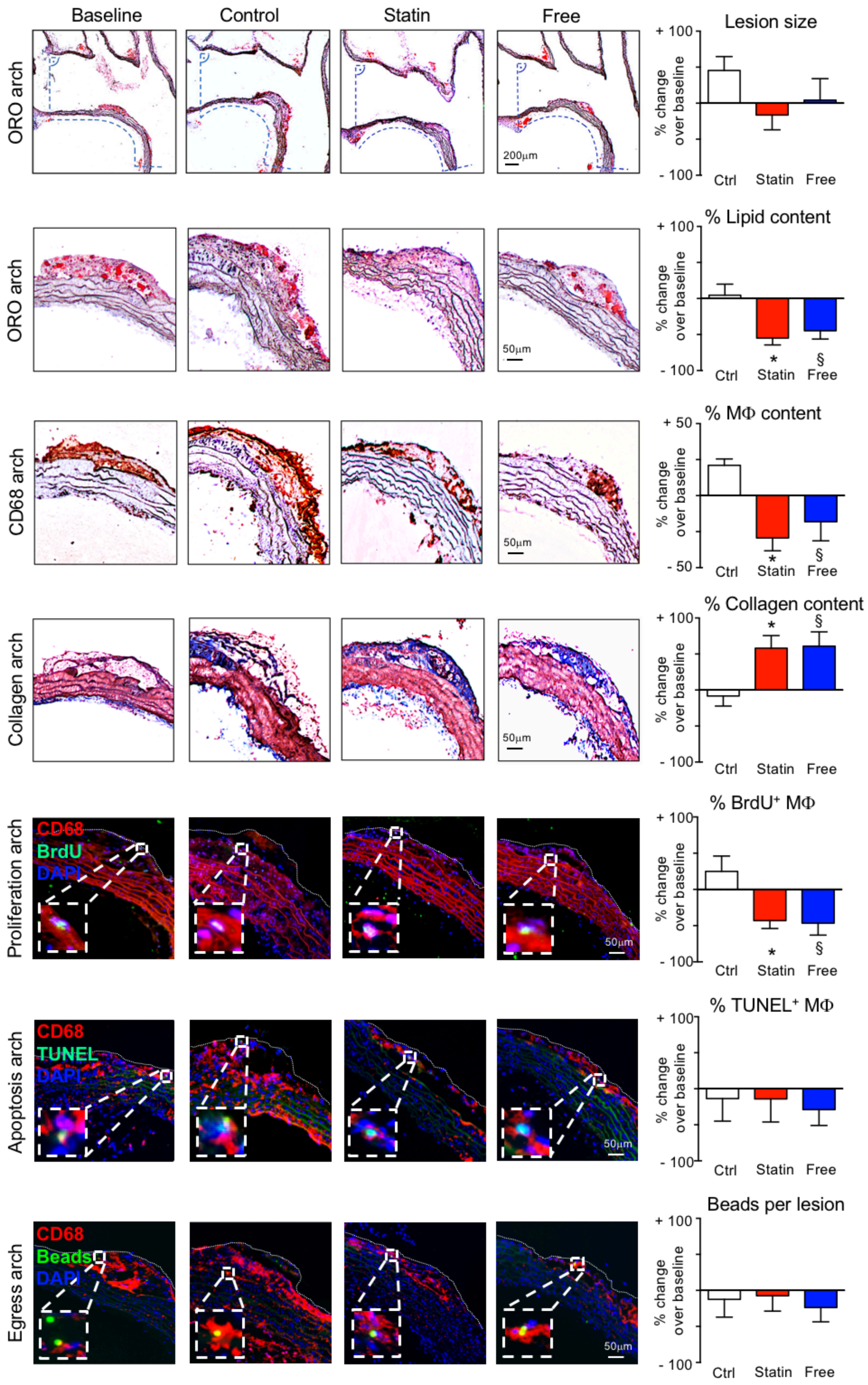

Supplemental Fig. 2. Serum cholesterol lowering leads to phenotypic plaque regression in aortic arches of APOE*3-Leiden.CETP mice. Representative images of aortic arch sections for all groups stained with Oil-red O (ORO, lipid), anti-CD68 (MΦ) and Masson's Trichrome (collagen), anti-BrdU (proliferation), TUNEL (apoptosis), and YG-fluorescent beads (egress) from top to bottom. Lesion size was quantified in a 2 mm long segment along the lower curvature of the aortic arch as indicated. On the

right, mean lesion area was determined in 3 aortic arch sections, and plaque composition was analyzed by quantifying the percent ORO^+, $CD68^+$ and $collagen^+$ areas within lesions. Proliferating MΦ were counted as $BrdU^+$ and $CD68^+$. Apoptotic MΦ were counted as $TUNEL^+$ and $CD68^+$. MΦ egress was estimated based on the change in numbers of fluorescent beads in lesions over time. These data are presented as mean ± SEM percent change over values in the baseline group to visualize relative changes during plaque progression and regression. *,§ $p<0.05$ denote statistically significant differences between the Ctrl and Statin (*) or Free (§) groups, baseline n=5-6, all other 3 groups n=8 per group, one-way ANOVA.

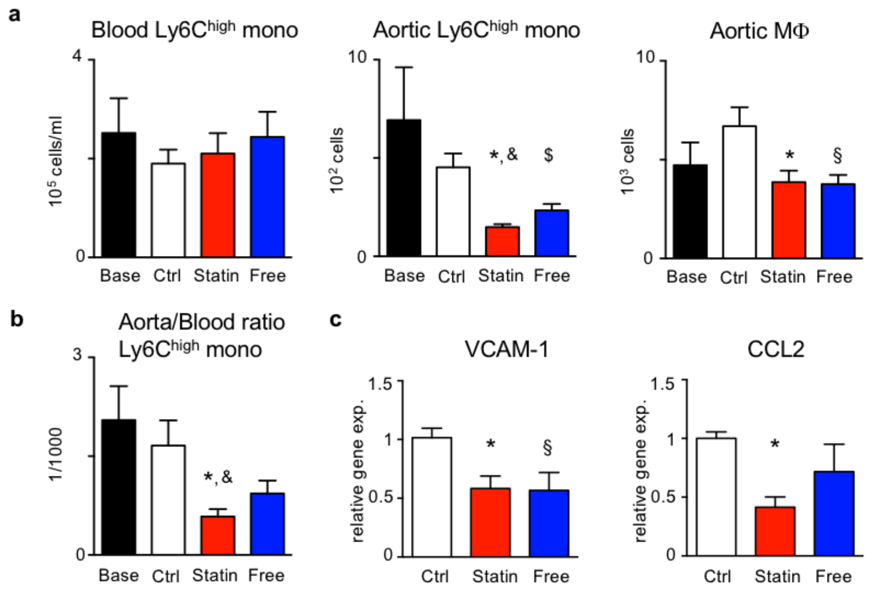

Supplemental Fig. 3. Atorvastatin treatment reduces monocyte recruitment to atherosclerotic aortas. (a) Flow cytometry-based quantification of $Ly6C^{high}$ monocyte and macrophage (MΦ) numbers in the blood and aortic cell suspensions. Results are presented as mean ± SEM, *,§ $p<0.05$ denote statistically significant differences between the Ctrl and Statin (*) or Free (§) groups, and between Base and Statin (&) or Free ($) groups, respectively, for baseline group n=12 (blood) and n=6 (aorta), for all other groups n=14 (blood and aorta) from 2 independent experiments, one-way ANOVA. **(b)** Intraindividual ratios of absolute $Ly6C^{high}$ monocyte counts quantified in aortic cell suspensions and blood (assuming 2ml of blood volume). Results are presented as mean ± SEM, * $p<0.05$ denote statistically significant differences between the Ctrl and Statin (*) or between Base and Statin (&) groups, for baseline group n=6, for all other groups n=14 from 2 independent experiments, one-way ANOVA. **(c)** VCAM-1 and CCL2 expression in atherosclerotic aortas. Results are presented as mean ± SEM fold change relative to control group values, *,§ $p<0.05$ denote statistically significant differences between the Control and Statin (*) or Free (§) groups, n=8 per group, one-way ANOVA.

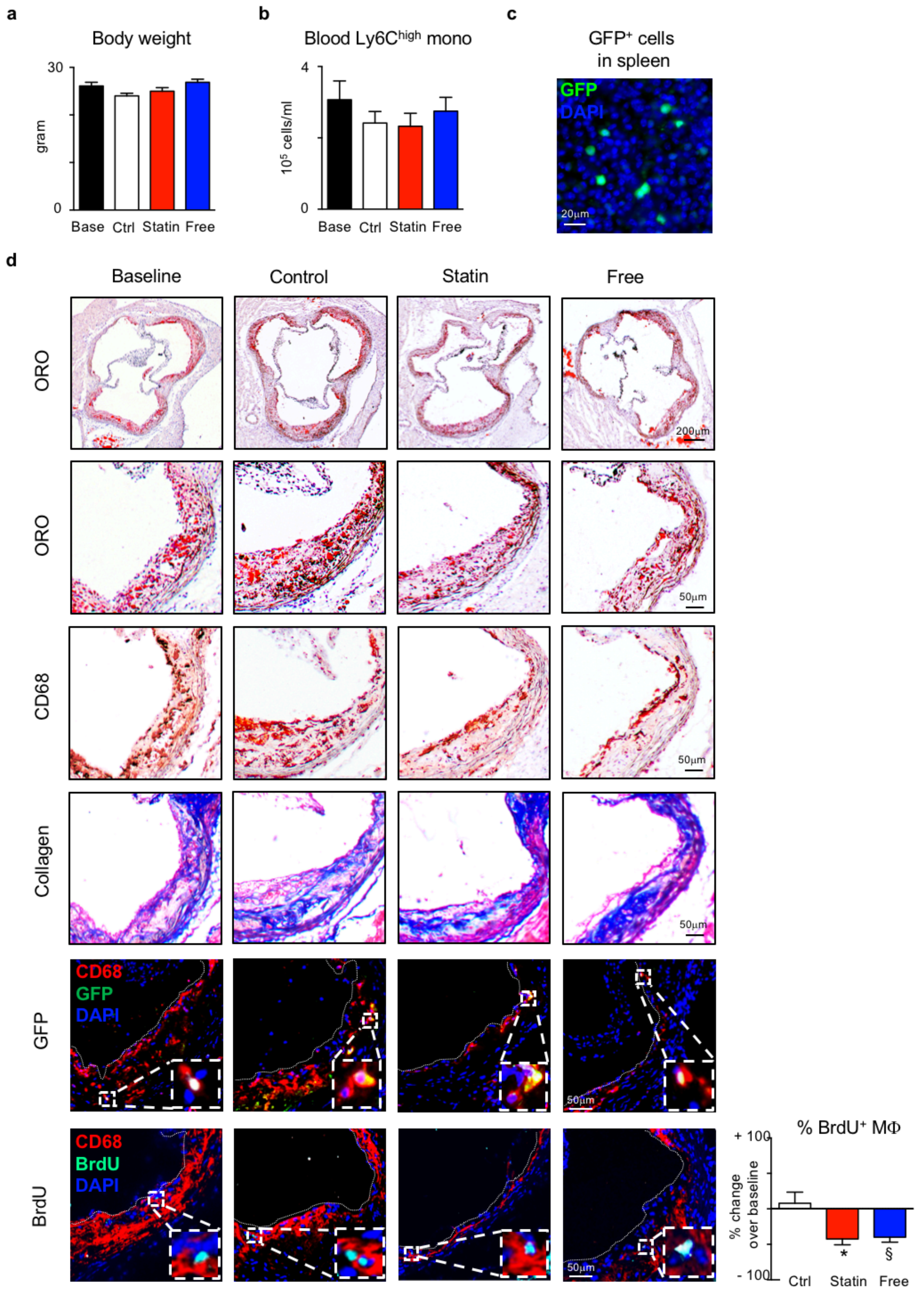

Supplemental Fig. 4. Serum cholesterol lowering leads to phenotypic plaque regression in shielded aortic roots of irradiated APOE*3-Leiden.CETP mice reconstituted with GFP^{+} bone marrow. (a, b) Body weights and flow cytometry-based quantification of Ly6C^{high} monocyte numbers in blood at time of sacrifice in APOE*3-Leiden.CETP mice after lethal irradiation with thoracoabdominal shielding and GFP^{+} bone marrow cell transplantation and randomization into 4 study groups (see Fig. 3a). Results are presented as mean ± SEM, baseline group n=12, other groups n=16 from 2 independent experiments, one-way ANOVA. **(c)** Exemplary image of GFP^{+} cells in the spleen 9 weeks post lethal irradiation with thoracoabdominal shielding and GFP^{+} bone marrow cell transplantation. **(d)** Representative images of

aortic root sections for all groups stained with Oil-red O (ORO, lipid), anti-CD68 (MΦ), Masson's Trichrome (for collagen), anti-GFP, and anti-BrdU. Quantitative results are presented in Figure 3C and on the right as mean ± SEM percent change over values in the baseline group. *,§ $p<0.05$ denote statistically significant differences between the Ctrl and Statin (*) or Free (§) groups, baseline n=6, all other 3 groups n=8 per group, one-way ANOVA.

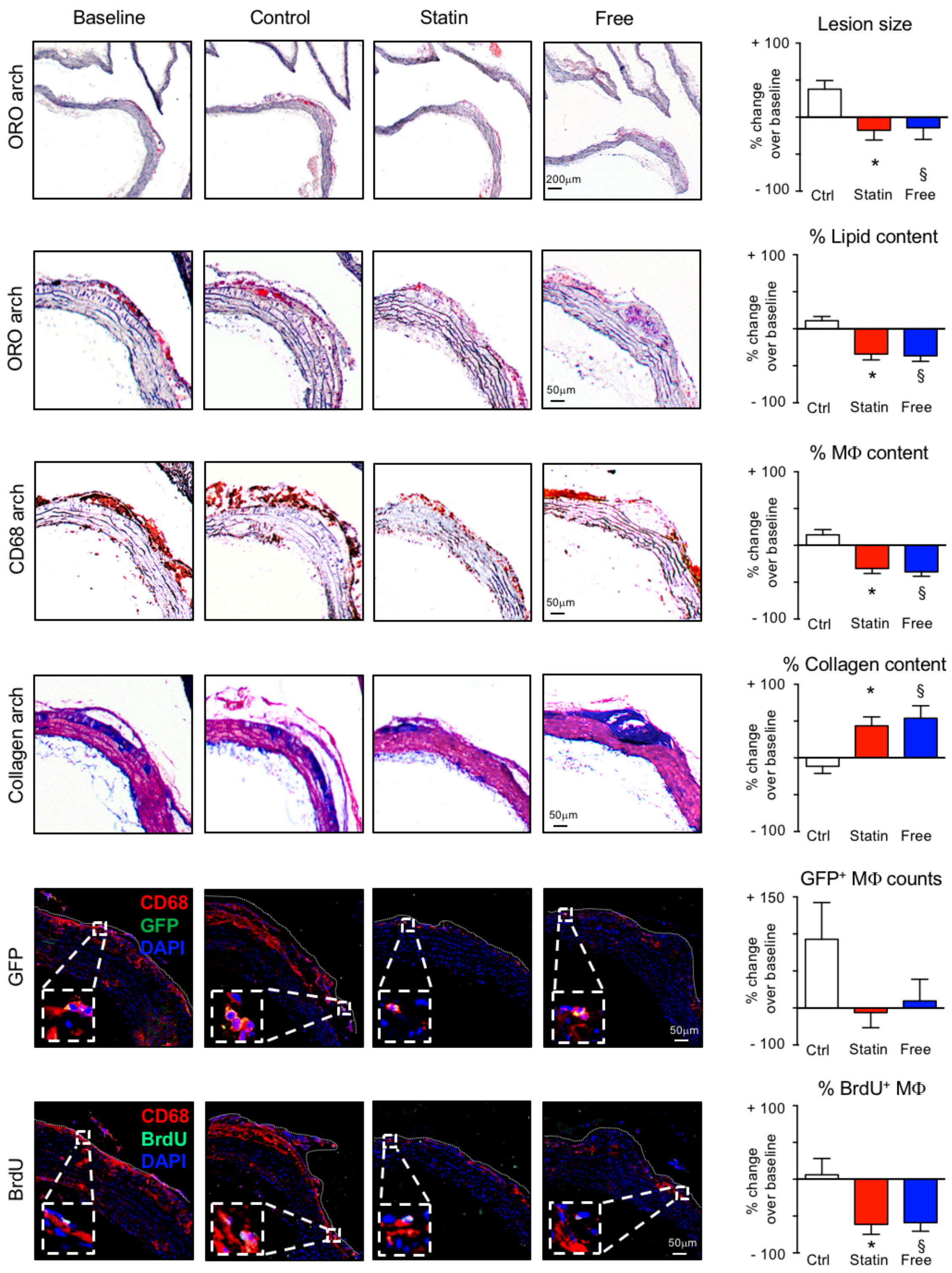

Supplemental Fig. 5. Serum cholesterol lowering leads to phenotypic plaque regression in shielded aortic arches of irradiated APOE*3-Leiden.CETP mice reconstituted with GFP^{+} bone marrow. Quantification of atherosclerotic lesion size and plaque composition in the aortic arch. On the right, results are presented as mean ± SEM percent change over values in the baseline group to visualize relative changes during plaque progression and regression. *,§ $p<0.05$ denote statistically significant differences between the Ctrl and Statin (*) or Free (§) groups, baseline n=6, all other 3 groups n=8 per group, one-way ANOVA. Representative images of aortic arch sections of all groups stained with Oil-red O (ORO, lipid), anti-CD68 (MΦ), Masson's Trichrome (for collagen), anti-GFP, and anti-BrdU are shown on the left.

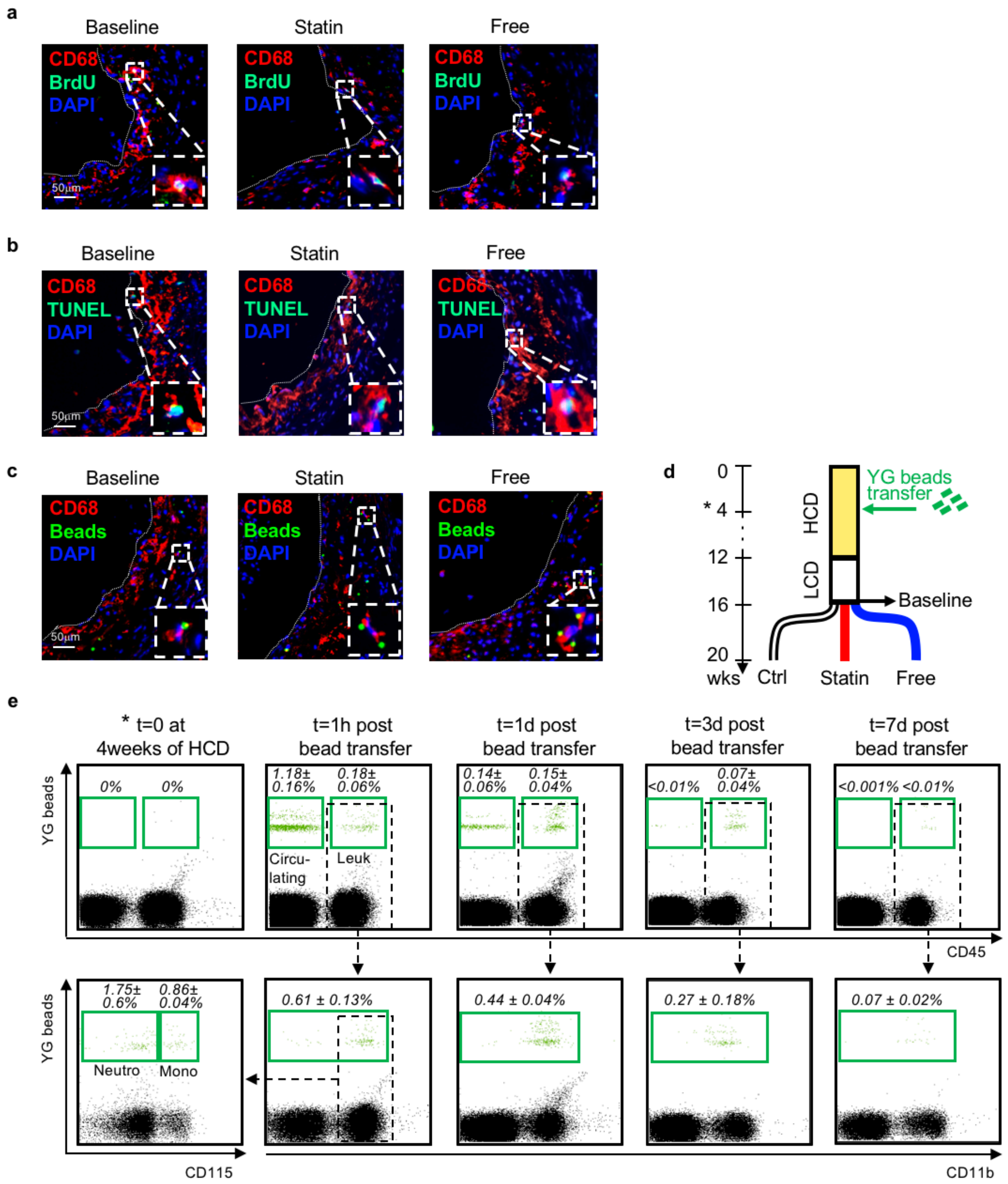

Supplemental Fig. 6. Fluorescent bead uptake by circulating myeloid cells. (a-c) Representative images of aortic root lesions stained for CD68, BrdU **(a)**, TUNEL **(b)**, and YG beads **(C)** for APOE*3-Leiden.CETP mice in the Base, Statin, and Free groups. **(d)** Study design and diet scheme in analogy to Fig. 1a modified by a single intravenous injection of YG beads at 4 weeks of high-cholesterol diet (HCD). **(e)** Flow cytometric quantification of YG beads in the blood circulating freely ($CD45^-$) and within leukocytes (Leuk; $CD45^+$) before and up to 7 days post intravenous injection. Monocytes (Mono) are identified as $CD11b^+$ $CD115^+$ and neutrophils (Neutro) as $CD11b^+$ $CD115^-$. Results are presented as mean percent ± SEM YG^+ fraction of the corresponding population.

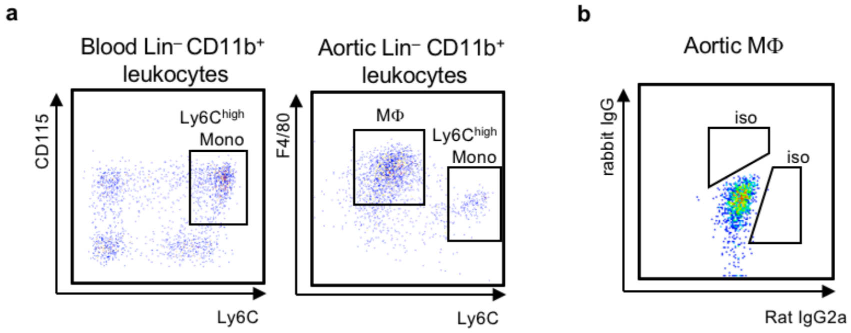

Supplemental Fig. 7. Flow cytometric identification of blood and aortic Ly6C^{high} monocytes (Mono) and macrophages (MΦ), and their proliferation. (a) Representative dot plots and gating strategies for identifying Ly6C^{high} monocytes (Mono) and macrophages (MΦ) in the blood and enzymatically digested aorta, respectively. **(b)** Representative dot plot showing intracellular isotype control staining for Ki67 and active Caspase 3 in aortic macrophages (MΦ).

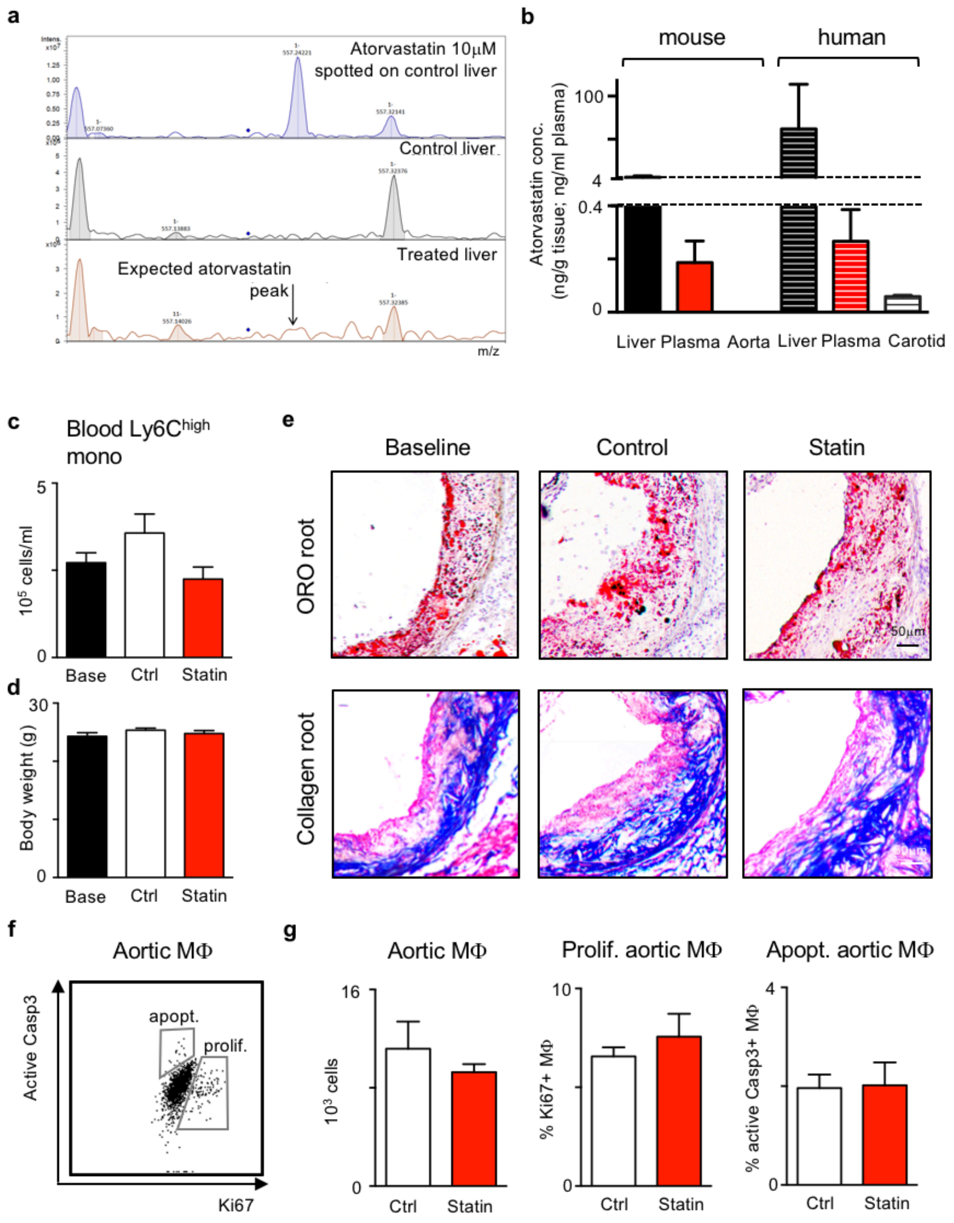

Supplemental Fig. 8. Atorvastatin acts primarily *via* cholesterol lowering in the liver. (a) Representative 7T MALDI-FTICR spectra (intensity vs mass-to charge (m/z)) of liver homogenates from LCD mice with (upper lane) and without (middle lane) atorvastatin added ex vivo for calibration and quantification as compared to liver homogenates from mice treated with oral atorvastatin (lower lane). **(b)** LC-MS/MS-based quantification of atorvastatin concentrations in livers, plasma, aortic and carotid artery tissue samples, respectively, of n=3 mice and n=3 humans. **(c)** Blood Ly6C^{high} monocyte counts were quantified by flow cytometry and manual counting. Results are presented as mean ± SEM, n=7 Apoe$^{-/-}$ mice after 12 weeks of high-cholesterol + 4 weeks of low-cholesterol diet at baseline (Base), and in n=12-13 Apoe$^{-/-}$ mice from 2 independent experiments fed a continued low-cholesterol diet (LCD) with (Statin) or without (Ctrl) atorvastatin for another 4 weeks, one-way ANOVA. **(d)** Body weights at baseline (Base) and following another 4 weeks of LCD with (Statin) or without (Ctrl) atorvastatin, results are presented as mean ± SEM, Base n=7, Ctrl and Statin, each n=12, from 2 independent experiments, one-way ANOVA. **(e)** Representative images of aortic root lesions for Base, Ctrl and Statin groups. **(f, g)** Aortic macrophage cell counts, and proportions of Ki67 and active caspase 3 co-staining quantified by flow cytometry. A representative dot plot is shown in **(f)**, results are presented as mean ± SEM, n=5 per group, Student's t-test.

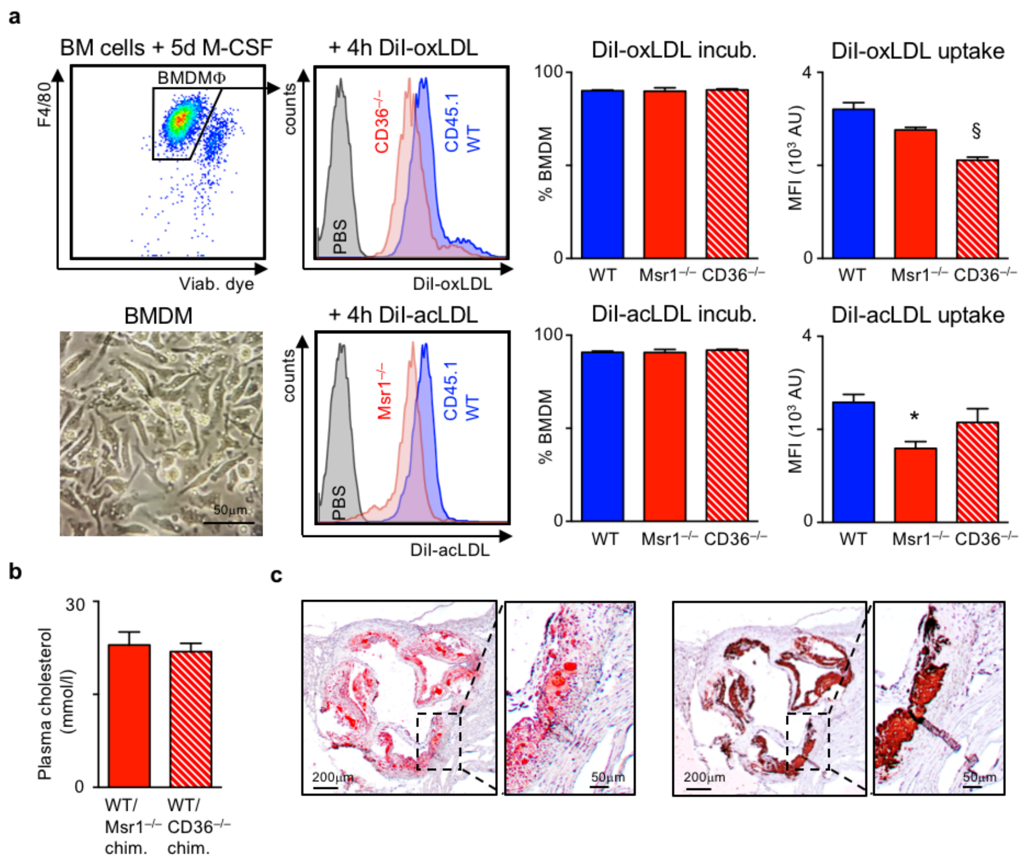

Supplemental Fig. 9. MΦ scavenger receptors Msr1 and CD36 mediate modified LDL uptake. (a) On the left, representative dot plot, microscopy image and histograms showing mean fluorescence intensity (MFI) of DiI-labeled medium oxidized (ox) or acetylated (ac) LDL within viable bone marrow-derived MΦ (BMDM) from CD45.1 WT, CD45.2 Msr1$^{-/-}$ or CD36$^{-/-}$ mice compared to PBS stimulation. On the right, quantification of differentiated viable BMDM in culture, and MFI of ingested DiI-oxLDL or DiI-acLDL in the 3 groups. Results are presented as mean ± SEM, *,§ $p<0.05$ denote statistically significant differences between the WT and Msr1$^{-/-}$ (*) or CD36$^{-/-}$ (§) groups, n=4 per group, Kruskal-Wallis. **(b)** Plasma cholesterol levels in Ldlr$^{-/-}$ mixed bone marrow chimeras (reconstituted with CD45.1 WT and CD45.2 Msr1$^{-/-}$ or CD36$^{-/-}$ bone marrow cells for 6 weeks) following 3 months of high cholesterol diet (HCD) feeding. **(c)** Representative images of aortic root sections stained with Oil-red O (left) and anti-CD68 (right) of Ldlr$^{-/-}$ mixed Msr1$^{+/+}$/Msr1$^{-/-}$ chimeras following 3 months of HCD.

Pat. No	Read-out	Sex (m/f)	Age (y)	Smoking history	Diabetes	Art. HTN	Statin, dose (mg)	LDL-C (mg/dl)
1	Histo	m	77		X	X	Atorva, 40	97
2	Histo	f	61			X	Eze/Simva, 10/40	84
3	Histo	m	79			X	Atorva, 80	52
4	Histo	m	50			X	Atorva, 40	154
5	Histo	m	85				Atorva, 80	52
6	Histo	m	67			X	None	139
7	Histo	m	62			X	Simva, 20	143
8	Histo	m	66	X			Atorva, 40	82
9	Histo	m	59	X		X	Simva, 80	39
10	Histo	m	77	X		X	Simva, 80	77
11	Histo	m	47			X	Atorva, 40	69
12	Histo	m	70	X		X	Atorva 40	68
13	Histo	m	56	X	X	X	Eze/Atorva, 10/80	113
14	Histo	f	76	X		X	Eze/Simva, 10/10	70
15	Histo	m	75	X	X	X	None	105
16	Histo	m	57		X		Atorva, 80	51
17	Histo	m	60				None	155
18	Histo	m	52			X	Atorva, 80	104
19	Histo	m	82			X	Atorva, 80	136
20	Histo	m	76	X		X	Simva, 20	161
21	MS	m	76			X	Eze/Atorva, 10/80	53
22	MS	m	86			X	Atorva, 80	50
23	MS	f	52	X	X	X	Atorva, 80	32

Supplemental Table 1. Characteristics of patients undergoing carotid endarterectomy. Plaque specimens of patients (Pat.) 1-20 were embedded for histologic staining (Histo), and plaques of patients 21-23 were processed for mass spectrometry (MS). 'X' marks positivity for cardiovascular risk factor. m, male. f, female. y, years. Art. HTN, arterial hypertension. Eze, ezetemib. Atorva, atorvastatin. Simva, simvastatin. LDL-C, serum low-density lipoprotein cholesterol level.

Pat. No	Read-out	Sex (m/f)	Age (y)	Smoking history	Diabetes	Art. HTN	Statin, dose (mg)	LDL-C (mg/dl)
24	MS	f	53	X		X	Atorva, 40	ND
25	MS	m	54	X	X		Atorva 40	ND
26	MS	f	64	X	X	X	Atorva, 80	ND

Supplemental Table 2. Characteristics of patients undergoing liver tissue sampling during bariatric surgery. MS, mass spectrometry. m, male. f, female. y, years. 'X' marks positivity for cardiovascular risk factor. Atorva, atorvastatin. ND, not determined.

Target antigen	Antibody clone	manufacturer	Dilution
Anti-CD3	145-2C11	Biolegend	1:500
Anti-CD11b	M1/70	BD Bioscience	1:300
Anti-CD19	eBio1D3	eBioscience	1:500
Anti-CD115	AFS98	eBioscience	1:300
Anti-CD45.1	A20	Biolegend	1:500
Anti-CD45.2	104	eBioscience	1:500
Anti-Caspase 3	C92-605	BD Bioscience	1:100
Anti-F4/80	BM8	BioLegend	1:300
Anti-Ki67	SolA15	eBioscience	1:300
Anti-Ly6C	HK1.4	Biolegend	1:500
Anti-Ly6G	1A8	Biolegend	1:500
Anti-NK1.1	PK136	Biolegend	1:500
Rabbit IgG	Poly4064	Biolegend	1:100
Rat IgG2a	G013C12	Biolegend	1:300

Supplemental Table 3. List of antibodies used for flow cytometry.
